# Why exercise builds muscles: titin mechanosensing controls skeletal muscle growth under load

**DOI:** 10.1016/j.bpj.2021.07.023

**Published:** 2021-08-10

**Authors:** Neil Ibata, Eugene M. Terentjev

**Affiliations:** 1 Cavendish Laboratory, University of Cambridge, Cambridge, United Kingdom

## Abstract

Muscles sense internally generated and externally applied forces, responding to these in a coordinated hierarchical manner at different timescales. The center of the basic unit of the muscle, the sarcomeric M-band, is perfectly placed to sense the different types of load to which the muscle is subjected. In particular, the kinase domain of titin (TK) located at the M-band is a known candidate for mechanical signaling. Here, we develop a quantitative mathematical model that describes the kinetics of TK-based mechanosensitive signaling and predicts trophic changes in response to exercise and rehabilitation regimes. First, we build the kinetic model for TK conformational changes under force: opening, phosphorylation, signaling, and autoinhibition. We find that TK opens as a metastable mechanosensitive switch, which naturally produces a much greater signal after high-load resistance exercise than an equally energetically costly endurance effort. Next, for the model to be stable and give coherent predictions, in particular for the lag after the onset of an exercise regime, we have to account for the associated kinetics of phosphate (carried by ATP) and for the nonlinear dependence of protein synthesis rates on muscle fiber size. We suggest that the latter effect may occur via the steric inhibition of ribosome diffusion through the sieve-like myofilament lattice. The full model yields a steady-state solution (homeostasis) for muscle cross-sectional area and tension and, a quantitatively plausible hypertrophic response to training, as well as atrophy after an extended reduction in tension.

## Significance

How intracellular signaling in muscle cells organizes a trophic response is a central question in exercise science, rehabilitation practice, and the study of muscle homeostasis (including development, aging, and numerous pathologies). Cells use time-integrated mechanical stimuli to initiate signaling cascades in a way that depends on the strength and duration of the signal. Our work provides a quantitative analytical rationale for a mechanosensitive mechanism for trophic signaling in muscle and gives evidence that the titin kinase domain is a good candidate for hypertrophic mechanosensing. We expect advances in targeted exercise medicine to be forthcoming, specifically if the exact structure of the mechanosensing complex bound to the TK domain and its downstream signaling cascade are studied in more detail.

## Introduction

Why does exercise build skeletal muscles, whereas long periods of immobility lead to muscle atrophy? The anecdotal evidence is clear, and the sports and rehabilitation medicine community has amassed a large amount of empirical knowledge on this topic. But the community has not as yet addressed and understood two key phenomena that underly hypertrophy and atrophy: how does the muscle “know” that it is being exercised (when it is certainly not the tactile sense, processed via the nervous system, that is at play in this), and how does it signal to provoke a morphological response to an increase or a lack of applied load? In some communities, there is a perception that muscle grows after exercise because of its internal repair of microdamage inflicted by the load. However, it is obvious that such an idea cannot be true for several reasons: most of the “tissue repair” occurs by growing connective tissue, whereas we need an increase of intricately hierarchical myofilament structure; also, this concept will not account for atrophy developing in microgravity or after extended bedrest.

Here, we develop a quantitative theoretical model that seeks to explain both of these processes. To be useful, the model must build on the relevant knowledge accumulated from studies of the anatomy and physiology of muscles, as well as the biological physics of molecular interactions and forces.

Muscles, their constituent cells, and the structure of their molecular filament mesh must respond mechanosensitively—i.e., in a manner that depends on the changes in the magnitude of the forces and stresses that arise during the contraction and extension of the muscle—at many different timescales. At the fastest timescales (tens or hundreds of milliseconds), skeletal muscles can produce near-maximal force for jumping or for the fight-or-flight response. Most muscles also go through cycles of shortening and lengthening with a period of the order of a second in the vast majority of sprint or endurance exercises (running, climbing, etc.) At a much longer timescale of many days, a muscle must also be able to measure changes in its overall use to effect adaptive muscle hypertrophy or atrophy, ultimately helping to prevent injury on the scale of months and years.

How the muscle cell keeps track of the history of its load and stress inputs within a number of intracellular output signals (which then go on to stimulate or inhibit muscle protein synthesis) is inherently an incredibly complex biochemical question. With the help of recent theoretical insights into the folding and unfolding rates of mechanosensor proteins under force, we hope to gain insights into the first part of this puzzle for the specific case of muscle hypertrophy. To make progress, we use a simple model for force-induced transitions between the different conformations of the titin kinase (TK) mechanosensor. If the conformational change helps create an intracellular signal, we can model the signal’s strength in terms of the duration and intensity of the mechanical inputs (external force on the TK domain in our case).

### Force chain

**Figure 1:**
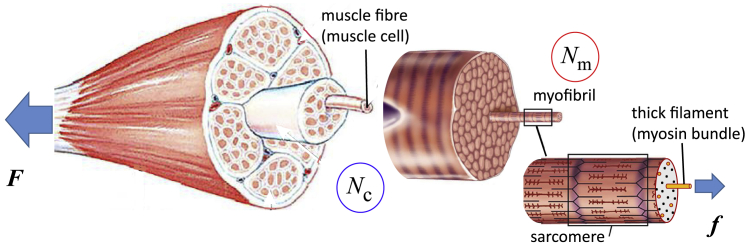
The “textbook” hierarchy in the anatomy of skeletal muscle. The overall muscle is characterized by its cross-sectional area (CSA), which contains a certain number (*N*_c_) of muscle fibers (the muscle cells with multiple nuclei or multinucleate myocytes). A given muscle has a nearly fixed number of myocytes: between *N*_c_ ≈ 1000 for the tensor tympani and *N*_c_ > 1,000,000 for large muscles (gastrocnemius, temporalis, etc. (1)). Muscle cells contain a variable number (*N*_m_) of parallel myofibrils (organelles), each of which can be divided into repeated mechanical elements called sarcomeres. The typical length of a sarcomere is ∼2 *μ*m, so there are ∼10^5^ of these elements in series along a fiber in a typical large muscle (2). Each sarcomere contains a number of parallel thick filaments (helical bundles of myosin, *red*) whose constituent myosins pull on the actin polymers in the thin filaments (F-actin, *blue*) to generate force. Within the myofibrils, the spacing between neighboring myosin filaments is ∼0.046 *μ*m at rest (3,4). The typical cross-sectional area of a single muscle fiber substantially varies between individuals and muscle types but is of the order of 4000 *μ*m^2^ (5). Accounting for some 15% of the cell volume being outside of the myofibrils (6), this means that a typical muscle fiber has ∼2,000,000 parallel filaments, between which the macroscopic force *F* must be divided. Rather than using this awkward number, we will express our results in terms of the total myofibrillar CSA within a single muscle fiber. A chemically activated muscle fiber with a CSA of 4000 *μ*m^2^ shows a force in the vicinity of 300–1000 *μ*N for untrained individuals (with a very large individual variation) (7), which translates to an average filament force of 150–500 pN (see Supporting materials and methods, Section A.6). Training can increase the neural activation level (8) as well as the number of active myosin heads and the maximal voluntary contraction force per filament (e.g., by stretch activation (9)). Because of this, we would expect resistance training to lead the filament forces to tend toward the upper end of the range (∼500 pN).

The individual subcellular, cellular, and supercellular components of a muscle act in concert to scale up a vast number of molecular force-generating events into a macroscopic force. The hierarchical structure of the muscle (see Fig. 1) allows the macroscopic and microscopic responses to mirror each other (10).

**Figure 2:**
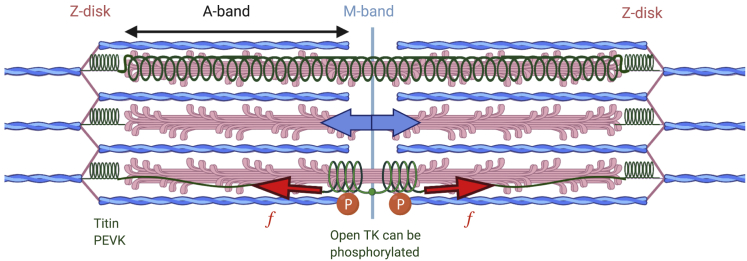
A sketch of the mechanically active elements of sarcomere. The thick filaments are cross-linked across the M-line, with six titin molecules bonded to these filaments on each side of the M-line. The full filament is under the measurable microscopic force identified in Fig. 1, shown by the blue arrow in the middle filament. At the molecular level, the force is borne by the individual titin and myosin filaments. If we assume that the thick filament and titin extend by the same amount during muscle contraction, then the graphical relationship between titin force and force in the thick filament is illustrated in Fig. S3. This figure illustrates an additional possibility: if titin wraps around the thick filament (*top*), then TK can lengthen substantially more than we consider in this work for titin extending with the thick filament (*bottom*). The force in TK would be much higher, making TK bear more load and create a greater mechanosensitive signal.

The sarcomere is the elementary unit of the muscle cell and the basic building block of the sliding filament hypothesis (11,12). Its regular and conserved structure, sketched in Fig. 2 for the vertebrate striated muscle, allows for a series transmission of tension over the whole length of the muscle. In vertebrates, six titin molecules are wrapped around each thick filament (13,14) on either side of the midpoint of the sarcomere: the M-line.

During active muscle contraction, myosin heads (motors) bind to actin and “walk” in an ATP-controlled sequence of steps (15) along the thin filaments. When resistance is applied, the myosin motor exerts a force against it. During slow resistance training in both concentric and eccentric motions, tension is passed along the sarcomere primarily through the thin filament, myosin heads (16,17), and the thick filament and into the cross-bridge region of the sarcomere where thick filaments are cross-linked with their associated M-band proteins.

The load in each of the sarcomere components ultimately depends on the relative compliance of elements. The relative load on the thick filament and the M-band segments of titin when the filament is either under internal (contracting) or external (extending) load is discussed in Supporting materials and methods, Section A.4. It is well known that titin is under load when the sarcomere is extended (18,19). Recent x-ray diffraction experiments (20) suggest that that the thick filament may be more compliant than originally thought; if so, M-line titin is likely substantially extended and loaded titin when the muscle actively generates force. Others disagree (21) and attribute the change in line spacing in diffusion experiments to a mechanosensitive activation of the entire thick filament at low forces. Either way, M-band titin is under some tension during active muscle contraction. This situation is sketched in Fig. 2.

We estimate the force in each filament both macroscopically and microscopically (see the full discussion in the Supporting materials and methods, Sections A.2 and A.3). We divide the force in the entire muscle by the number of active myofilaments (see Fig. 1) to find a large variation in force per filament in untrained individuals (150–500 pN) (7). Muscle fiber neuronal and molecular activation increases with training (8,9), so the higher forces are more likely representative of filament forces in trained individuals. The maximal filament forces extrapolated from x-ray diffraction studies (22) are higher at ∼600 pN, possibly because the actin only partially binds to myosin in normal contractions, maximal forces do not last very long, and the muscle does not coordinate perfectly as a whole. In the Supporting materials and methods, Section A.4, we graphically find an approximate relation between titin and thick filament force. In particular, it suggests that the force per titin be close to 25 pN at the maximal voluntary contraction force.

In Section A.1 of the Supporting materials and methods, we discuss different candidates of mechanosensitive signaling in the sarcomere and highlight the reasons why titin kinase is a particularly good candidate for this role and why we have not considered some of these other candidates here.

### TK is a mechanosensor of the “second kind”

Cells sense and respond to the mechanical properties of their environment using two main classes of force receptors. The first type of mechanosensor responds immediately under force (23,24). Mechanosensitive ion channels are the archetypal example of such a sensor and have been proposed to play a role in tactile signaling (transforming a mechanical signal into chemical) (23,25). However, the ions that they use in signaling are rapidly depleted, making it difficult for these sensors to signal in response to a sustained force.

The other type of mechanosensor, dubbed of the “second kind” by Cockerill et al. (26), can either indirectly “measure” the response coefficients or time integrate an external force acting on the molecule. The focal adhesion kinase (FAK) mechanosensor (27,28) is a good example; it can sense substrate stiffness by measuring the tension in the integrin-talin-actin force chain, which binds a cell to its extracellular matrix. FAK and the TK domain both open under force, can be phosphorylated, and appear pivotal to mechanosensitive signaling of the second kind; they also have many structural similarities. TK has already been suggested to act as a mechanosensor (29, 30, 31), and although recent experimental work has focused mainly on other regions of the titin molecule, we believe that it is worth returning to the TK domain to examine it as a time-integrating mechanosensor. In the Results below, we see that the metastability of the TK open state, when the muscle is under steady-state passive tension, can indeed allow for the TK domain to help produce increased signal levels long after the end of an exercise session.

### TK domain opens under force

Many signaling pathways use a molecular switch to initiate a signaling cascade. One of the most common post-translational modifications of proteins involves the reversible addition of a phosphate group to some amino acids (mainly tyrosine); this addition alters the local polarity of the target protein, allowing it to change its shape and bind a new substrate (32). Phosphorylation can form the basis for signaling if an input changes the protein’s conformation, from a native folded conformation that cannot bind to a phosphate group (often called “autoinhibited”) to an “open” conformation in which the geometry of the molecule allows phosphate groups to be donated to the phosphorylation site (27). The phosphorylated protein can then bind to a third substrate molecule and can either directly catalytically affect or indirectly activate a signaling pathway.

Protein unfolding under force has been analyzed extensively, beginning with studies of the titin Ig domain (33,34). These experiments show characteristic force-extension curves, which can help deduce the transition energies between conformations for the molecules in question. We note that the Ig domains unfold under quite a high force (33,35,36) and could initially appear to be candidates for mechanosensors too. However, very few phosphorylation sites have been found on the Ig domains compared with the remainder of the molecule (37), suggesting that they do not contribute to force-induced signaling, but rather help control the length of the titin molecule and avoid immediate sarcomere damage under high load.

Titin kinase was initially thought to be the only catalytic domain on titin (38). Bogomolovas et al. (39) suggest that TK acts as a pseudokinase, simply scaffolding the aggregation of a protein complex when it is phosphorylated and allowing for another protein to be allosterically phosphorylated. Computational and experimental studies of TK have shown that its force-length response also follows a characteristic stepwise unfolding pattern, but with much smaller steps than those observed for the Ig domains. In particular, atomic force microscopy (AFM) experiments (29) show that the presence of ATP (an energy supply) changes the conformational energy landscape of the molecule as it is stretched. This shows that the molecule possesses a long-lasting open conformation of its TK domain, in which it can accommodate the recruitment of signaling molecules upstream of a mechanosensitive signaling pathway before the protein unfolds completely and potentially loses its signaling ability. Being the largest known molecule in vertebrates, titin interacts with an unsurprisingly large number of molecules (40,41); Linke et al. (42) summarized this knowledge in a protein-protein interaction network, shown in their Fig. 2, in which in particular the nbr1 and MuRF pathway (localized in the M-band) is shuttled into the nucleus, leading to SRF and transcription of new actin.

## Materials and methods

### Methods used in modeling

We model a resistance training repetition as a piecewise function for force. During the loading phase (start at *t* = 0), the force increases from the initial force *f*(*t* = 0) and asymptotically approaches the maximal force per filament *f*_max_ during the repetition with a rate *k*_*f*_ ≈ 30 s^−1^. The full-muscle rate of force development is substantially lower at ∼5 s^−1^ (43), but we assume that there is a lag due to the macroscopic muscle providing some slack before macroscopic force development. It therefore seems likely that the molecular rate of sarcomere force development (which impacts the rate of titin being placed under force) is closer to the much faster rate of force increase during muscle tetani. During the unloading phase, the muscle force decreases with a fast rate (the same rate as force development for tetani, a bit slower for twitches, but ultimately insignificant relative to the timescales of repetition). The force per titin, as well as the muscle opening and closing rates *k*_−_ and *k*_+_, is calculated at every time step. Because the TK conformations quickly change during exercise, the next time step of the numerical integration is adaptively calculated at each time step as a fraction of the greatest fractional change in all of the molecular species in the model. Several repetitions make up a set, and several sets make up an exercise session. The exercise regime is assumed to be adaptive such that the repetition force on TK remains constant as the total myofibrillar cross-sectional area (CSA) increases.

### The model

**Figure 3:**
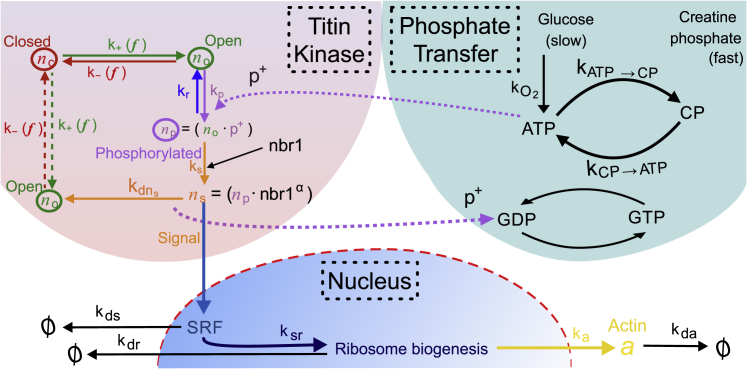
Sketch of the kinetic processes that link titin kinase opening and phosphorylation, mechanosensing complex formation, signal activation, ribosome biogenesis, and the increased synthesis of structural proteins (of these, only actin is listed for simplicity).

Here, we explain why we believe that the kinetic processes schematically shown in Fig. 3 are the necessary elements for any TK-based treatment of mechanosensing of the second kind and of subsequent mechanosensitive intracellular signaling. Our model can be divided into three parts:

1. The opening and phosphorylation of the TK domain. This stage is highly nonlinear because TK opens as a mechanosensitive switch and because the mechanosensitive complex binds allosterically. The open state is metastable if the muscle is under a steady-state load.
2. The creation and degradation of signaling molecules, new ribosomes, and structural proteins. All of these rates can be approximated as linear, apart from a size feedback term, which arises because ribosomal diffusion is sterically hindered in large cells (see discussion below).
3. Exercise can only be so hard before the muscle depletes its short-term energy supplies. The balance between energy generation from oxidative phosphorylation and the depletion of short-term energy stores has to be considered to correctly model the dynamic response.

#### Opening and phosphorylation of TK domain

The energy barrier for the transition between the “closed” native domain conformation and the “open” conformation that supports ATP binding and phosphorylation is the key determinant of the kinetic transition rates between the two TK states. AFM data collected by Puchner et al. (29) are essential here; we match the relevant TK conformations to their data and explain how to extract several important model parameters in Sections A.4 and A.5 of the Supporting materials and methods.

In the absence of any signaling, the concentration of total (free + bound) ATP is constant, and the transitions from closed to open to phosphorylated TK domain conformations are simple and reversible.

1. Closed ↔ open: TK can open under force with a force-dependent rate constant *k*_+_(*f*) and likewise close with a force-dependent rate constant *k*_−_(*f*). Here, we use the framework of (28) to derive these two rate constants. The concentrations of the closed and open conformations are *n*_c_ and *n*_o_, respectively; cf. Fig. 3.
2. Open ↔ phosphorylated: the open state of TK can be phosphorylated with a rate constant *k*_*p*_; the total rate of this process depends on both the concentration of ATP and of the open TK, [ATP] and *n*_o_. The phosphorylated state with the concentration *n*_p_ can also spontaneously dephosphorylate with a rate constant *k*_*r*_ but cannot spontaneously close until then.

This cyclic reaction, illustrated in the TK section of Fig. 3 is described by the kinetic equations for the evolution of *n*_c_, *n*_o_, and *n*_p_:

(1) $$\frac{dn_{\text{c}}}{dt}=-k_{+}n_{\text{c}}+k_{-}n_{\text{o}},$$

(2a) $$\frac{dn_{\text{o}}}{dt}=k_{+}n_{\text{c}}-k_{-}n_{\text{o}}-k_{p}n_{\text{o}}\left[\text{ATP}\right]+k_{r}n_{\text{p}},$$

(3a) $$\frac{dn_{\text{p}}}{dt}=k_{p}n_{\text{o}}\left[\text{ATP}\right]-k_{r}n_{\text{p}},$$

and

(4a) $$n_{\text{c}}+n_{\text{o}}+n_{\text{p}}=n_{\text{titin}}\left(\text{constraint}\right),$$

where the last condition encodes the total concentration of TK units; this is equal to the concentration of titin and remains constant on the timescale of signaling. These equations are examined in the Supporting materials and methods, Section A.6, in which they are shown to adequately reproduce the phosphorylation kinetics of TK measured by Puchner et al. (29), providing an a posteriori justification for their use.

#### Signal generation from phosphorylated TK

The phosphorylated TK domain can bind the zinc finger domain protein nbr1 (44) and begin to form an aggregate; the concentration of the signaling complexes *n*_*s*_ must be introduced with a new separate kinetic equation. The mechanosensing complex identified in the most general formulation by Lange (44) is a multispecies aggregate, which we consider in more detail in the Supporting materials and methods, Section A.7.

SRF, the mechanosensitive signaling molecule in the Lange model, is known to undergo activation by phosphorylation (45,46). There are many phosphorylation sites on nbr1 and p62, and some on MuRF (47), which suggests that SRF could be activated by phosphate transfer originating from TK. An activation would most likely irreversibly alter the conformation of the signaling complex and result in the disassembly of the complex every time a new signaling molecule was activated. Assuming that the complete mechanosensing complex has a time-independent probability to disassemble, with a rate $k_{dn_{s}}$*n*_*s*_, we estimate the corresponding rate constant $k_{dn_{s}}$ from experiments (48) that show the increase in phospho-SRF (activated signal) after exercise. They find that the level of activated SRF binding to DNA increases by a factor of 2 an hour after skeletal muscle cell contraction and reaches half of its maximal increase after 10 min of exercise. This means that the degradation rate of the mechanosensing complex occurs with a half-life of ∼10 min ($k_{dn_{s}}$ ≈ 1/600 s^−1^).

We can now rewrite our kinetic equations to add the formation and degradation rates of the signaling complex, as well as the activation of the SRF signal, to Eq. 1:

(2b) $$\frac{dn_{\text{o}}}{dt}=k_{+}n_{\text{c}}-k_{-}n_{\text{o}}-k_{p}n_{\text{o}}\left[\text{ATP}\right]+k_{r}n_{\text{p}}+k_{dn_{s}}n_{s},$$

(3b) $$\frac{dn_{\text{p}}}{dt}=k_{p}n_{\text{o}}\left[\text{ATP}\right]-k_{r}n_{\text{p}}-k_{s}n_{\text{p}},$$

(5) $$\frac{dn_{s}}{dt}=k_{s}n_{\text{p}}-k_{dn_{s}}n_{s},$$

(4b) $$n_{\text{c}}+n_{\text{o}}+\left(n_{\text{p}}+n_{s}\right)=n_{\text{titin}}\left(\text{constraint}\right),$$

and

(6) $$\frac{dn_{\text{SRF}}}{dt}=k_{dn_{s}}n_{s}-k_{ds}n_{\text{SRF}}.$$

The concentration of ATP is expressed in number per titin; the total phosphate is assumed to scale proportionately to the size of the myofibril and the number of titin molecules. In the Supporting materials and methods, Section C, we also track the kinetics of ATP depletion during intense exercise. The additional equations are mathematically more complicated and do not help understand the full model but are included in the numerical simulations in the Results.

These are the core equations that describe the relatively fast activation of a signaling molecule during muscle loading. We show in the Results below that they display a very pronounced switching behavior; in other words, small changes in tension result in large changes to the signal concentration. We also find that these equations support an increase in the concentration of signal (possibly SRF) for a substantial time of the order of a couple of days, which could help account for the immediate increase in protein synthesis postexercise. But we shall see in the next section that a simple one-step signal cannot by itself account for the observed time dependence of hypertrophy.

#### Muscle protein synthesis after mechanosensor signaling

The constituent molecules of most signaling pathways have a short lifetime relative to that of the structural proteins. It is also well documented that a few bouts of exercise do not have a tangible effect on muscle volume and that muscle takes at least a few weeks to begin to show visible hypertrophic adaptations. The debate on whether true hypertrophy is soon detected or whether initial postexercise changes in muscle CSA are the signs of muscle microdamage is a rather fraught one (49, 50, 51, 52). Three weeks of resistance training appears to be a consensus time, after which true hypertrophy is actually detected. This means that there has to be a way of “integrating” the signal over such a long period of time, beyond the scope of the simple force integration supported by a metastable open state of TK. Here, we combine the above model of mechanosensitive signaling with a simple model of protein synthesis from a signaling molecule and propose a mechanism by which this integration may occur.

Based on a review and discussion of the current literature in the Supporting materials and methods, Section B.2, we conclude that it is likely an increase in ribosome biogenesis (rather than the temporary increase in mRNA transcript number) that allows for this “time integration” of the signal. Its effect would be to suppress fluctuations in the concentration of TK conformations or signaling molecules, smoothly increasing the concentration of the structural muscle proteins over the time similar to the half-life of ribosomes. We suggest that this effect could help explain the delay of a few weeks between starting resistance exercise and the first detection of measurable muscle growth, as noted by trainers and rehabilitation specialists.

New experiments show that sarcomeric proteins are synthesized in situ at the sarcomeric Z-line and M-band (53). As far as we are aware, ribosomal subunits can only move by diffusion, whereas mRNA can be actively transported to the synthesis site. The inhibition of the diffusion of ribosomal subunits by the myofilament lattice (54) could reduce the synthesis of new sarcomeric proteins by a sizeable amount (5–10%) in adult myocytes. The fractional reduction in titin synthesis can be written in the form −*αn*_titin_, where the coefficient *α* depends on the ribosome diffusion constant, the lattice spacing, and the rate of lysosomal degradation. This term has several important consequences; it provides a bound on muscle growth or shrinkage, and it affects the speed of muscle size adaptations. We examine this point in more detail in the Supporting materials and methods, Section B.4.

We use the number of titin molecules *n*_titin_ in the muscle fiber cross section as a proxy for the muscle fiber CSA because the hierarchical sarcomere structure is well conserved in most muscles at rest. When necessary, one can convert from one to the other, as in Fig. 1. Eqs. 1, 2b, 3b, 4b, 5, and 6 are combined with the following equations (more details in the Supporting materials and methods, Section B):

(7) $$\frac{dn_{\text{rRNA}}}{dt}=k_{sr}n_{\text{SRF}}-k_{dr}n_{\text{rRNA}}$$

and

(8) $$\frac{dn_{\text{titin}}}{dt}=k_{st}n_{\text{rRNA}}\left(1-\alpha n_{\text{titin}}\right)-k_{dt}n_{\text{titin}}.$$

In the Supporting materials and methods, Section E, we consider the possibility that the force produced by the muscle does not scale linearly with muscle size. It is unclear exactly how much active muscle force scales with muscle size. Krivickas et al. (7) find that force increases slower at larger muscle CSA, whereas Akagi et al. (55) do not see a substantial nonlinearity between force and myofiber volume. So, in the main body of this work, we proceed with the simplest assumption of the linear scaling.

## Results

The steady-state load required for the muscle to maintain homeostasis can be obtained analytically. Once we have “zeroed” our problem by checking that this value makes sense in terms of steady-state tension (muscle tone) in the Supporting materials and methods, Sections B and C, we consider the dynamics of Eqs. 1, 2a, 2b, 3a, 3b, 4a, 4b, and 5 for TK only to show that it does indeed open as a metastable mechanosensitive switch. After that, we will proceed to study what effects different types of resistance exercise have on muscle fiber CSA and compare them with reports from the literature.

### Steady state

The steady-state solution to Eqs. 1, 2a, 2b, 3a, 3b, 4a, 4b, 5, 6, 7, and 8 is obtained in the Supporting materials and methods, Section D. We find the following tension per individual TK domain:

(9) $$f=\frac{\text{Δ}G_{0}}{u_{\text{max}}}+\frac{k_{B}T}{u_{\text{max}}}\text{ln}\left(\frac{\left(k_{r}+k_{s}\right)}{k_{p}\left[\text{ATP}\right]\left(\zeta -1-\frac{k_{s}}{k_{dn_{s}}}-\frac{k_{r}+k_{s}}{k_{p}\left[\text{ATP}\right]}\right)}\right),$$

where the shorthand *ζ* is the ratio of synthesis/degradation coefficients:

(10) $$\zeta =\frac{k_{st}\left(1-\alpha n_{\text{titin}}\right)k_{s}k_{sr}}{k_{dt}k_{dr}k_{ds}}.$$

**Figure 4:**
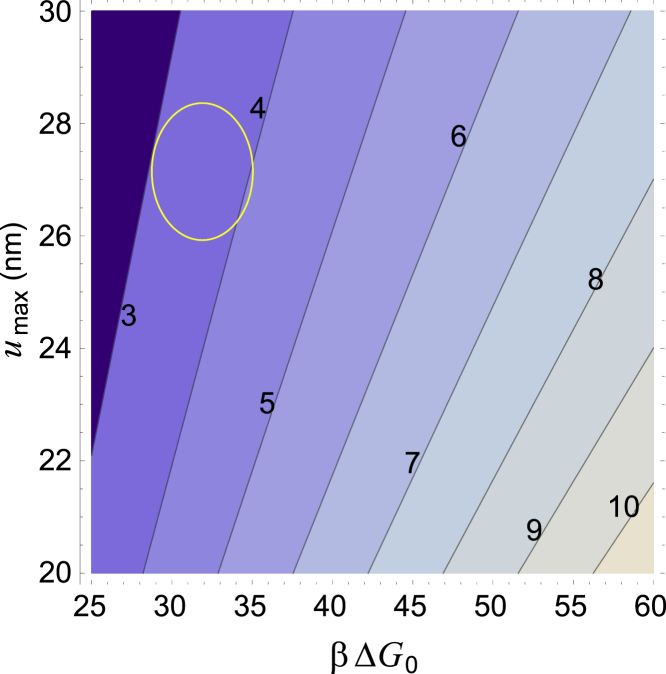
Steady-state force (expressed in piconewtons, labeled in *contour lines*) from (9) as a function of the TK activation energy Δ*G*_0_ and the opening distance of the mechanosensor *u*_max_. Δ*G*_0_ is expressed in dimensionless units scaled by the thermal energy *β* = 1/*k*_*B*_*T*, with *T* = 310 K. The values of rate constants are given in Table 1, and the following typical concentrations were used: *p*^+^ = 2000 per titin, nbr1_st_ = 0.1 per titin, and *σ* = 0.5. The circle marks the “sweet spot” in which the likely values of *u*_max_ and Δ*G*_0_ should be. Note that both Δ*G*_0_ and *u*_max_ are fixed physiological values, as is the maximal steady-state force. We do not have precise values for any of these (see Supporting materials and methods, Section A.5 for a more detailed explanation of the uncertainty in Δ*G*_0_), so it is still instructional to plot our model’s prediction of the steady-state force for different plausible values of the other two constants. To see this figure in color, go online.

The first key result here is that the force on the TK domain, which maintains a steady-state muscle fiber CSA, is determined almost exclusively by two parameters: the energy barrier Δ*G*_0_ between the closed and open conformations of the TK domain, and the unfolding distance *u*_max_. It is clear that changing any of the coefficients in the logarithm in Eq. 9 would only have a minor effect on the steady-state force. The typical resting muscle forces are plotted in Fig. 4 as a function of Δ*G*_0_ and *u*_max_ (illustrated in Fig. S5). The typical homeostatic force experienced by a TK domain is of the order of 2–10 pN.

**Figure 5:**
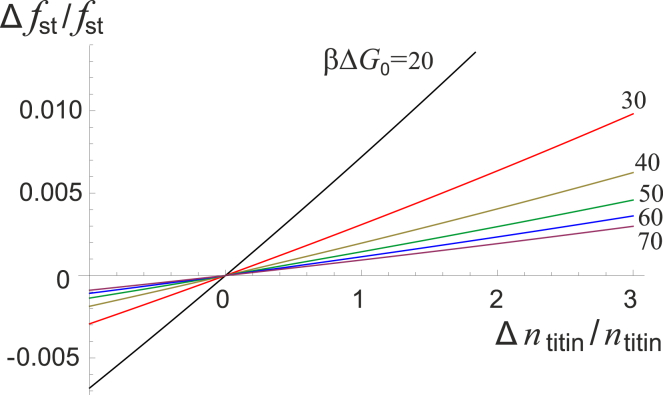
Fractional change in steady-state muscle force (*vertical axis*) versus fractional change in muscle size (*horizontal axis*), from (9). Note that Δ*n*_titin_ = −*n*_titin_ (the *left limit* of the axis) represents a complete degradation of the muscle. The values of opening energy Δ*G*_0_ are labeled on the plot. The values of rate constants are given in Table 1, and the following typical concentrations were used: *p*^+^ = 2000 per titin, nbr1_st_ = 0.002 per titin, and *σ* = 0.5. The maximal opening distance of TK was taken as *u*_max_ = 27 nm. The values for Δ*G*_0_ and *u*_max_ were estimated from AFM data and molecular dynamics simulations conducted by Puchner et al. (29) in the Supporting materials and methods, Section A.5. To see this figure in color, go online.

The other key point is that a small change in the muscle steady-state force (perhaps supported by an increase in tendon tension, which lengthens the sarcomeres) can maintain a large change in muscle size. The fractional change in the steady-state muscle tone as a function of the fractional change in muscle size is plotted in Fig. 5.

Combining Supporting materials and methods, Sections A.2 and A.3 (maximal thick filament force) and Section A.4 (titin force in terms of thick filament force), we estimate the force per titin during a contraction at the maximal voluntary contraction (MVC) to be∼25 pN. In the low-load regime, there is very little active muscle force (otherwise known as muscle tone), perhaps only 1–2% of the MVC force (56) (at most ∼1–2 pN per TK if titins were to bear most of the load), which matches well with the relative oxygen consumption in resting muscle (57). When sarcomeres operate at their optimal length, a non-negligible passive tension is developed by, among other effects, the extension of titin (58). In this regime, most of the load originates from this baseline stretch in the sarcomeres; indeed, Whitehead et al. (59) found that passive tension at the optimal sarcomere length was of the order of 5–10% of the MVC force. The passive tension value is much more consistent with our estimate for the resting tension per titin in the steady state (see Fig. 4).

Note that the passive force in the resting sarcomere can be substantially dialed by changing the stiffness of titin; the increased tension of the resting muscle would allow it to adjust to resistance training much more readily. The titin stiffness slowly diminishes after exercise, but the temporary increase in stiffness could also contribute to the “time integration” of the mechanosensitive signal. This complication is beyond the scope of our model.

### Titin kinase as a metastable mechanosensitive switch

**Figure 6:**
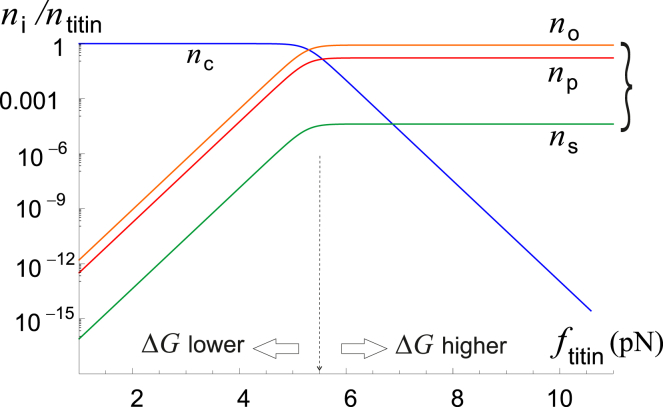
Log plot of the steady-state concentration of the TK conformations (*blue*, closed; *red*, open; *orange*, phosphorylated; *green*, bound to the mechanosensor complex) as a function of the steady-state force per titin from (9). As the steady-state force increases, the preferred conformation of TK switches from closed to a fixed ratio of open/phosphorylated/signaling complex bound. This plot is for Δ*G*_0_ = 35*k*_*B*_*T*. Note that the molecule switches from being preferably closed to preferably open/phosphorylated/signaling slightly above the steady-state force of a few piconewtons. But even though the steady-state conformation may be favored at forces even slightly above the resting muscle tension, titin takes a long time to open enough to actually signal in large numbers because the opening rate *k*_+_ is much less than 1 s^−1^ at low and medium forces (see Fig. 7 for an illustration of this behavior). To see this figure in color, go online.

**Figure 7:**
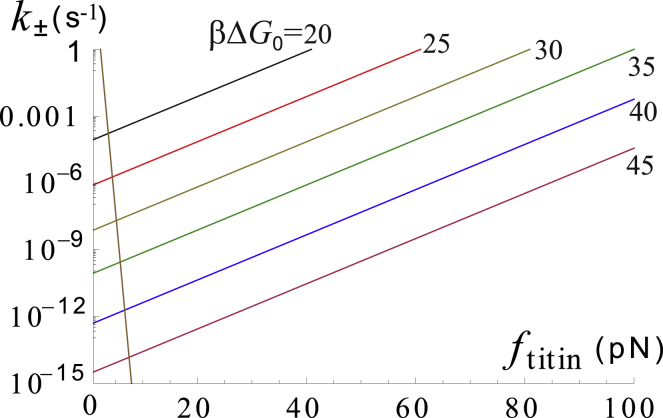
Log plot of the closing rate *k*_−_ (*brown*) and opening rates *k*_+_ for different values of activation barrier Δ*G*_0_, as labeled on the plot. Even when TK opening is favored, at *k*_+_ > *k*_−_, the opening rates are much less than 1 s^−1^, meaning that TK opens linearly with increasing time under load and exponentially with increasing force. We suggest that this behavior is the basis for high-intensity resistance training; doubling the force increases mechanosensitive signaling by several orders of magnitude. To see this figure in color, go online.

In Fig. 6, we see that TK obeys switching kinetics: above a critical load, its closed conformation is no longer favored. However, the low TK opening and closing rates *k*_+_ and *k*_−_ plotted in Fig. 7 do not allow TK to quickly change between its conformations at physiological loads. If resistance exercise increases the number of open TKs, their number will remain elevated up to days after exercise; in other words, the TK open/phosphorylated/signaling complex-bound state is metastable. We use numerical simulations to explore this point further in the next section.

TK signaling increases linearly with exercise duration (barring the effects of fatigue), whereas opening rates (and signaling) increase exponentially with force in TK. Although TK force scales roughly linearly with myosin force (see Fig. S3), this allows mechanosensitive signaling to increase much faster than the corresponding energetic cost at high exercise force. At very high forces, however, it appears that TK force increases much more slowly than myosin force, leading to a plateau in the efficiency of mechanosensitive signaling (Fig. S4). Excluding mechanosensitive signaling at the steady-state force (which is efficient because thick filament force is low but does not do much to change muscle CSA), signaling in response to resistance training is always more effective as the load increases until at least about ∼70% of the MVC force. Our model does not extend to how muscle fatigue induces changes in muscle stiffness (60,61), which could alter TK signaling kinetics at high forces as well.

### Long-term mechanosensitive signaling and response

To compare our model with experimental data in the literature, we consider a “typical” resistance exercise session consisting of three sets of 10 repetitions (more details in the Materials and methods section above). This mimics a common resistance training program (see, e.g., DeFreitas et al. (49), who set up resistance training sessions with 8–12 repetitions to failure over three sets). Choosing a specific value of repetition force is not straightforward because although most force studies consider MVC force, most hypertrophy programs compare the training load to the single-repetition maximal load for a given exercise. The muscle force during one full repetition is necessarily smaller than the instantaneous force. Determining the corresponding force per TK might be further complicated because titin is under more load when the muscle is stretched (passive force) than when it is actively contracting. Nevertheless, our choice of 20 pN per titin seems to be supported by several factors discussed here and in the Supporting materials and methods.

**Figure 8:**
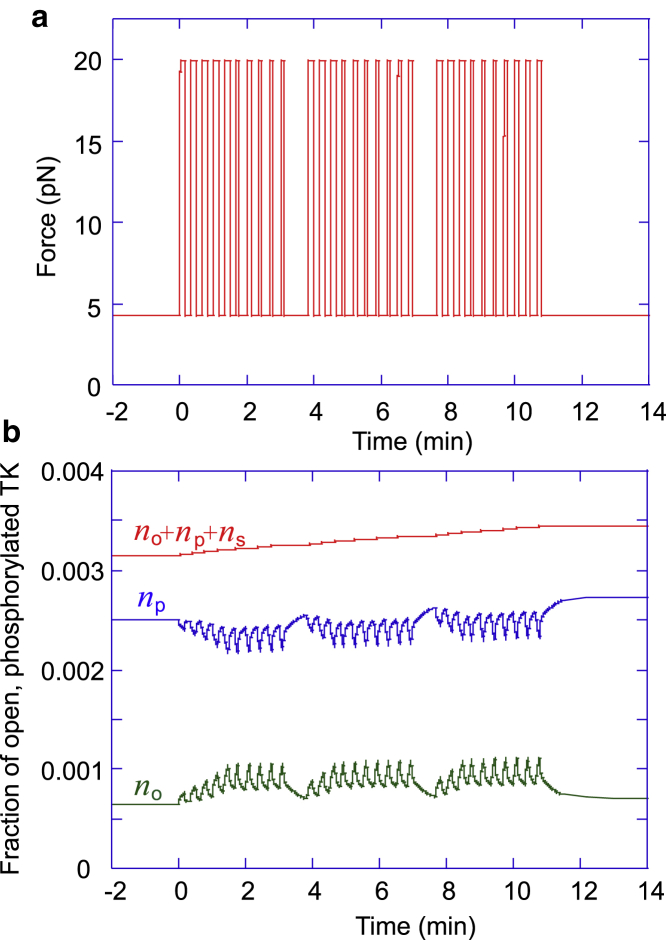
Simulation of an exercise session involving three sets of ten 10-s repetitions. (*a*) All repetitions are performed at the same force per titin, but their duration is cut short upon reaching exhaustion. As the number of titins increases, we assume that the training regime adapts by proportionately increasing the repetition force. (*b*) The depletion of ATP leads to a temporary drop in phosphorylated TK during exercise. However, the sum of open, phosphorylated, and signaling complex-bound TK steadily increases during the exercise. Because the closing rate of TK is quite low (of the order of 10^−5^ s^−1^, depending on the number of attempts at crossing the energy barrier and the barrier height Δ*G*_0_), the baseline concentrations of phosphorylated and signaling TK conformations remain elevated after exercise. To see this figure in color, go online.

We simulate a typical exercise session as a fixed number of repetitions at a given force, grouped into a fixed number of sets, as shown in Fig. 8 *a* (more details in the Materials and methods). During each repetition, the opening rate *k*_+_ of TK becomes much greater than its closing rate, which decreases the proportion of closed TK and increases its propensity to signal. Because the muscle is under a combination of passive and active tension at rest, the closing rate of titin is small after exercise, even though it is greater than the opening rate (see Fig. 7). This allows TK to revert to its steady-state conformation after a time of the order of hours to days, in a manner that depends on the number of attempts at crossing the energy barrier between the closed and open conformations (see Supporting materials and methods, Section A.5), as well as the height of the activation barrier Δ*G*_0_. The metastability of the open state at steady-state tension would then naturally allow the muscle to produce a mechanosensitive signal long after the end of exercise. This might account for the increase in myofibrillar protein synthesis in the 2 days after exercise, specifically resistance training (62,63).

**Figure 9:**
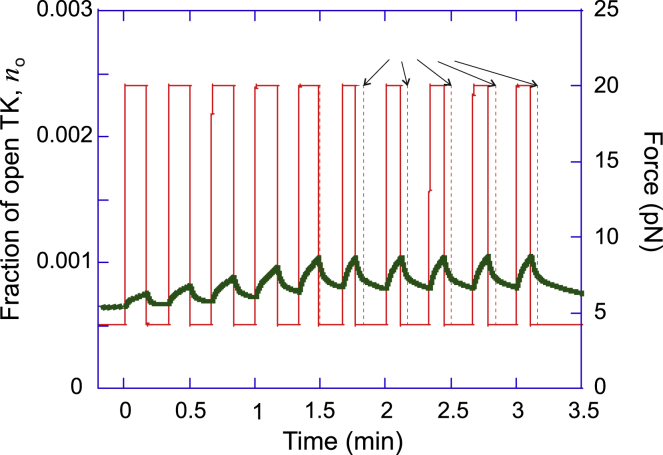
The first set of ten 10-s repetitions from Fig. 8. Note that the repetitions become shorter as ATP runs out during the period of high load; as the ATP level falls below a critical value (which we set to a half of the homeostatic level), the muscle can no longer sustain the load, and the only possibility is to drop the weight and return to the steady-state force. So, the period of loading becomes shorter than the prescribed period, shown in a dashed line in the plot and arrows marking the prescribed period. To see this figure in color, go online.

The important aspect of exercise, naturally reflected in our model, is the effect of ATP depletion. To make it clearer, we plot the same data as in Fig. 8, zooming in to just one (the first) set of repetitions in Fig. 9. Both myosin motors increase their ATP consumption under the high load, and the freshly open TK domains require ATP for phosphorylation. During the high-intensity loading, the level of ATP could drop below a critical value, after which the muscle would no longer be able to maintain the force: the only option is to drop the weight and return to the steady-state force recovery stage. We see that this effect occurs after a few repetitions in Fig. 9. We also find, in this simulation of model exercise, that subsequent sets of repetitions have this cutoff (driven by ATP depletion) of the later loading periods becoming less pronounced because the overall level of ATP marginally increases during the session.

ATP/creatine phosphate recovery between repetitions and sets is driven by speed at which the body can perform glycolysis, which in turn is dependent on the oxygen uptake rate (64). The rate of aerobic ATP resynthesis can be linked to the oxygen uptake per unit muscle volume (VO_2_max/L, the maximal uptake velocity, is a well-reported physiological parameter). This process is only ∼40% efficient (65) and accounts for much of the mechanical inefficiency in muscle (which has been reported as between 20 and 50% (66), depending on the stage of exercise. These necessary parameters are included in the model, which is expanded in the Supporting materials and methods, Section C. Viscous loss in muscles, basement membranes, fascia, and tendons likely accounts for a substantial part of the remaining energy dissipation (67, 68, 69), in a manner that depends on muscle length (70). However, if we assume that the internal energy dissipation in the thick filament-titin superstructure is minimal compared to the other sources of viscous energy dissipation (e.g., by filaments moving relative to their nearest neighbors), the force produced by the individual myosin motors during concentric muscle action will be directly related to the tension in the titin kinase domain. Furthermore, very recent work (71) suggests that viscous dissipation forces in the sarcomere are relatively minimal compared with active myosin force. Because of the inherent difficulty with any analysis of dissipative forces of this kind, we simply consider the microscopic TK tension to be directly proportional to the forces developed by the myosin motors attached to that filament.

**Figure 10:**
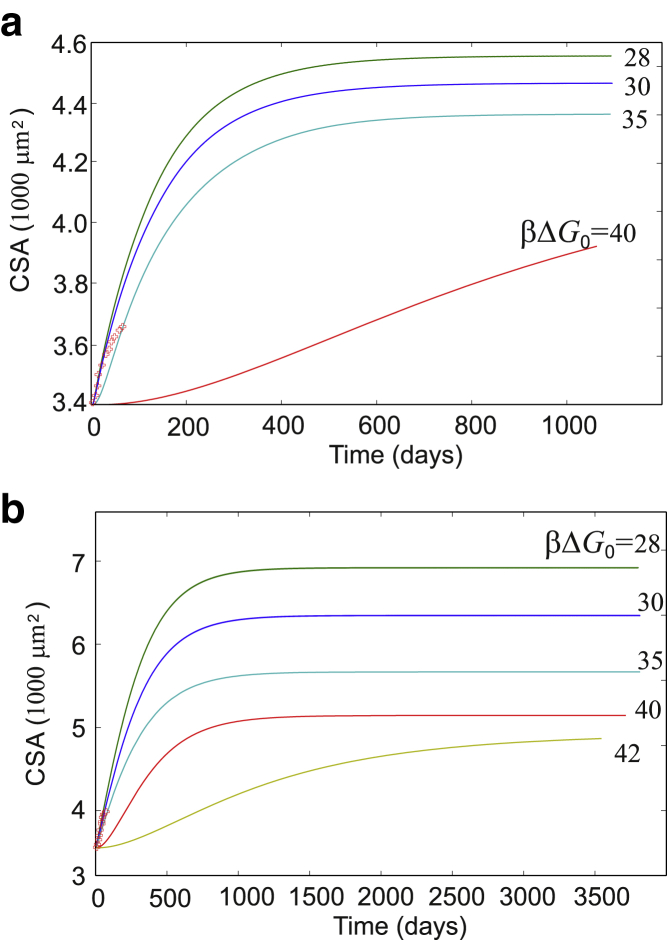
Time course of muscle growth in response to a regular resistance training program (exercise of Fig. 8, every 3 days). (*a*) The total muscle load *F* is kept constant, so the force per titin *f* effectively diminishes as the CSA increases. (*b*) The force per titin *f* is maintained constant (20 pN, as discussed before), which effectively implies that the total muscle load *F* increases in proportion with CSA (*vertical axis*). Several curves for different values of the energy barrier Δ*G*_0_ are labeled on the plot. As might be expected, muscle CSA changes are faster and greater in magnitude if the energy barrier Δ*G*_0_ is smaller (i.e., TK opens faster during exercise, and signals to a greater extent). We overlay the predictions of our model with measurements of fractional changes in muscle CSA over an 8-week period, measured by De Freitas et al. (49) (*red crosses*, same values in both plots). An initial force per titin of 20 pN matches well with real data, showing an ∼1% growth per week. To see this figure in color, go online.

**Figure 11:**
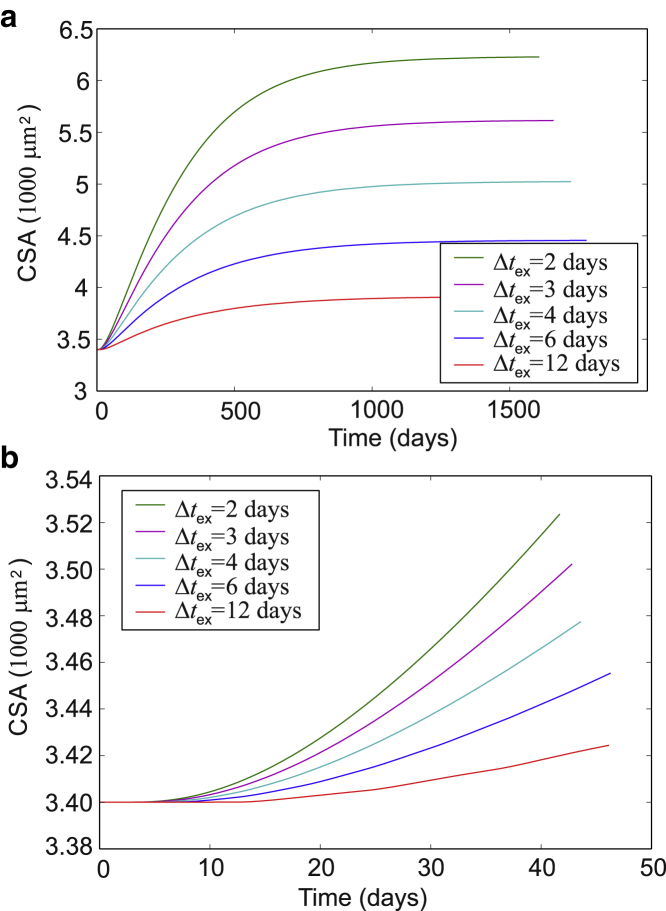
Time course of muscle response for different exercise frequencies. Here, we take *β*Δ*G*_0_ = 35 (see Fig. 10) and a representative value of ribosome diffusion inhibition *αn*_titin_ = 0.1 (see discussion in Fig. 12 for further details). (*a*) Full duration of the simulation: the total myofibrillar CSA asymptotically tends to a steady state after a few years. (*b*) The onset of muscle hypertrophy lags the start of the exercise regime by about a week because TK opening rates are slow and the signal is “integrated” by a combination of SRF and ribosomes. The initial rate of change of the total myofibrillar CSA can be compared with experiments, which show ∼1% CSA changes per week in response to high-intensity resistance exercise (72). This simulation shows a similar rate of CSA change, which means that a TK maximal force of ∼20 pN during high-intensity resistance exercise could produce an adequate signal for muscle hypertrophy to occur. Because of the switch-like nature of TK, it is unlikely that this maximal load on TK could be too different from 20 pN. This force value is consistent with a picture in which the myosins bear most of the load during active muscle contraction and titin acts as a parallel stretch sensor. To see this figure in color, go online.

In Figs. 10 and 11, and afterward, we return to measuring the muscle “size” directly by the total myofibrillar CSA (by converting to that from the measure of titin molecules, which is equivalent but carries less intuitive appeal). Because the volume of a myonuclear domain is close to 16,000 *μ*m^3^ and remains conserved in a developed adult muscle (73) and the density of titins is also an approximate constant (∼3000 per *μ*m^3^; see Fig. 1)—or an alternative equivalent estimate, the density of titins across the unit area of CSA (∼6000 per *μ*m^2^)—it allows quantitative measure of CSA as our output.

Also note that because in this test, we are applying a constant force per titin and the CSA increases with time, this means that the actual exercise load to the whole muscle must be increasing proportionally (in our simplified model, the relation between CSA and *n*_titin_ is linear) to achieve the optimal growth.

In Fig. 10, we test the long-term consequences of a regular resistance training program (the standard model exercise as in Fig. 8 *a*, repeated every 3 days). Several curves are presented, showing the final homeostatic saturation level, and the time to reach it, dependent on the key model parameter: the energy barrier Δ*G*_0_ for TK opening. The earlier discussion based on the data obtained by Puchner et al. (29) and the structural analogy between TK and FAK (27,74) suggest that Δ*G*_0_ could be around 30*k*_*B*_*T* (or ∼75 kJ/mol).

The comparison between plots in Fig. 10, *a* and *b* is important. As our model relies on the value of force per titin *f*, the total load on the muscle is distributed across filaments in parallel across the CSA. So, if one maintains the same exercise load, the effective force per titin diminishes in proportion to the growing CSA, the result of which is shown in Fig. 10 *a*. In contrast, one might modify the exercise by increasing the total load in proportion with CSA; Fig. 10 *b* shows the result of such an adaptive regime. In the nonadaptive case, the final saturation is reached in about a year, and the total CSA increase is ∼30% (assuming Δ*G*_0_ = 30*k*_*B*_*T*). In the adaptive exercise, the final saturation is reached much slower, but the total myofibrillar CSA increase is ∼88%, almost doubles the myofibrillar component of the muscle volume in ∼2 years time. It is reassuring that the experimental measurement of De Freitas et al. (49) of CSA growth over a period of 8 weeks, in a similar exercise regime, quantitatively agrees with our prediction of ∼1% CSA increase a week in the initial period.

The regularity of the exercise has a strong effect; the long-term magnitude of hypertrophy predicted by the model is affected by what happens on the daily basis. Fig. 11 *a* compares the long-term results when the interval between the model exercise Δ*t*_ex_ varies from frequent to very sparse bouts (the Δ*t*_ex_ = 3 days case in Fig. 10). We find that the extent of muscle hypertrophy is roughly linearly dependent on the exercise frequency.

We have seen that the TK mechanosensor can increase the rate of signal activation for an extended period of time after exercise. But this signal does not directly correlate with protein synthesis in the immediate aftermath of exercise. In particular, there is a known lag between the start of an exercise regime and the detection of muscle hypertrophy (51,52). This lag can be accounted for in our model if ribosomes are the main factor limiting an increase in protein synthesis and must be made more abundant before hypertrophy can occur (see Fig. 11 *b*). The remainder of the results section uses the full model, which includes SRF (signaling), ribosomes, and titin number.

### Adaptations to resistance training exercise

We showed in section 2 above that constant titin kinase mechanosensing at the steady-state muscle tension allows the muscle to maintain its size. To consider dynamic changes in muscle size, we must first assure ourselves that it reaches a new steady state; secondly, that it predicts that muscles grow with the correct time dependence; and finally, we must check whether the model predictions for the magnitude of change in muscle size are in a reasonable range, given that we have no free parameters (all rate constants and concentrations are independently known).

**Figure 12:**
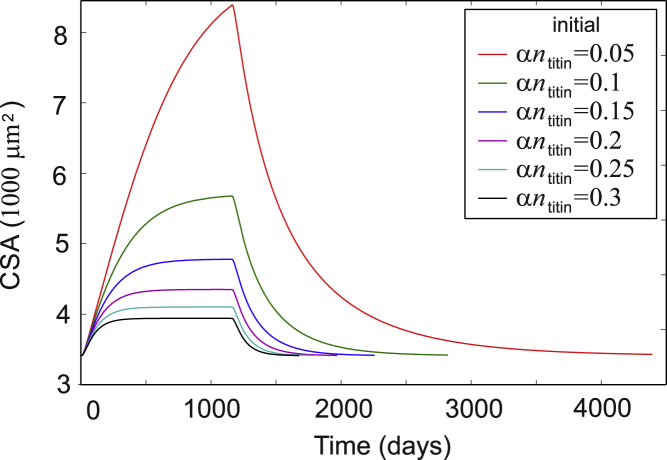
Time course of muscle growth and loss (starting after 600 days of hypertrophy) in response to a regular resistance training program (every 3 days) with three sets of 10 repetitions at 20 pN per titin (our estimate of ∼70% 1RM), followed by detraining. The diffusive feedback depends on the degree of sarcoplasmic titin degradation, which in turn increases with myonuclear domain size and lysosomal activity. Slow detraining may combine with an initial fast loss due to atrophic conditions (see below). In this case, a 5–10% ribosome degradation en route to the titin synthesis sites (0.05 < *αn*_titin_ < 0.1) appears to support training and detraining at the correct rates; see (8,49,75,76). To see this figure in color, go online.

In Fig. 12, we see that both muscle growth during the exercise program and muscle detraining after exercise program ends are strongly dependent on the feedback from the slow diffusion of ribosomes across the large and sterically hindered sarcoplasm. Greater muscle fiber CSA at the start of training implies more ribosomal diffusion blocking, hence a higher hindrance term *αn*_titin_, resulting in a faster, lower-magnitude response to the same training load. This behavior is qualitatively observed in the literature; strength-trained athletes respond to a much lesser degree to a resistance training regime (see, e.g., (72)).

After stopping a resistance training program, muscle CSA slowly decreases, eventually returning to its pretrained homeostatic value. The time course of detraining is harder to investigate. Low values of 2 months (8,75) for skeletal muscle to several years for recovering hypertrophic cardiac muscle (76) have been reported. In our model, we observe reasonable time courses that match this range for detraining for a 5–10% degradation of ribosomes before they arrive at the sarcomere or for very low force feedback in the range of 0.001 < *μ* < 0.005 (see Fig. 12).

There are some exceptions to this; career athletes maintain significantly higher muscle CSA a long time after retiring (77), and the body maintains a memory of prior resistance training events (78) by changing its methylome. It seems likely that the body can develop and maintain a higher resting muscle tone if chronic resistance training changes the molecular architecture of the muscle. This complication is beyond the scope of our model.

### Atrophy and recovery from bedrest or microgravity

When the body is subjected to bedrest, microgravity (79), famine (80), or as the consequence of several pathologies (81), muscle size can very rapidly decrease. Any mechanism that increases degradation rates (SRF, ribosomes, and titin degradation rates in our model; see Table 1) will necessarily cause atrophy, and our model confirms this (see Supporting materials and methods, Section E for detail).

**Table 1:** Values of rate constants directly obtained in experiments or simulations or extrapolated from the data presented

Constant	Value (s^−1^)	Source
*k*_*p*_	0.07 M^−1^	(29)
*k*_*r*_	6	(29)
*k*_*s*_	10^−8^–10^−6^	(82)
$k_{dn_{s}}$	0.002	(44,48)
*k*_*ds*_	10^−5^	(83)
*k*_*st*_	10^−5^	(84, 85, 86)
*k*_*dt*_	4 × 10^−6^	(87)
*k*_*sr*_	0.1	(88)
*k*_*dr*_	∼9 × 10^−7^	(89,90)

**Figure 13:**
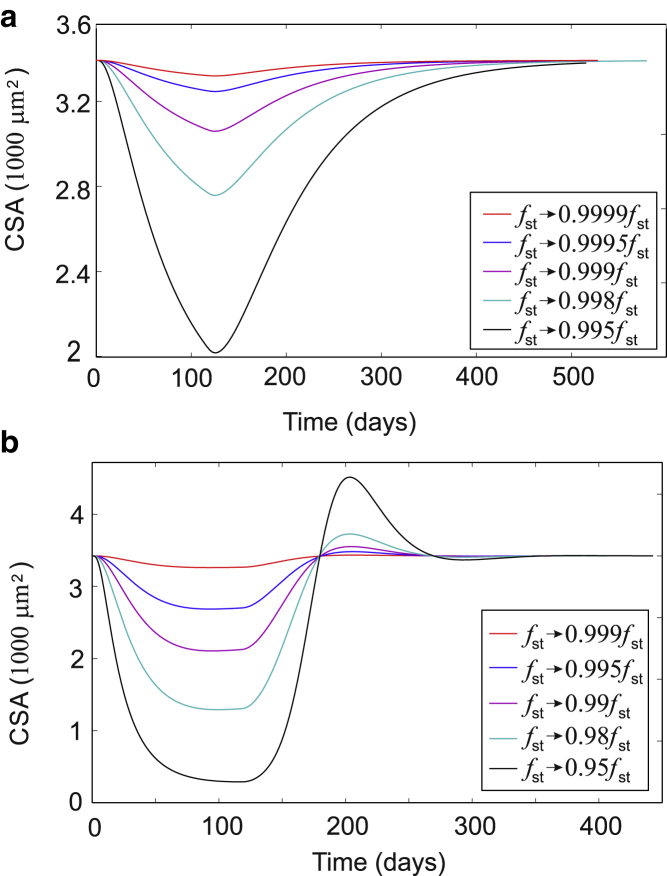
Time course of muscle atrophy as the steady-state force *f*_st_ (discussed in (9) and Fig. 4) is suddenly diminished from the steady-state value to a lower value. In this simulation, after 120 days, the force is brought up to its steady-state value again. The recovery speed depends on exactly how the muscle force scales with muscle CSA during atrophy, the “force feedback” discussed in Supporting materials and methods, Section B.4. (*a*) The case of negligible force feedback (*μ* = 0.005) leads to unphysiologically slow rates of atrophy and recovery over several months. (*b*) Higher force feedback (*μ* = 0.02) leads to much more reasonable recovery rates, which we consider close to clinical observations. To see this figure in color, go online.

Extended periods of bedrest and microgravity are the more interesting atrophy-inducing conditions to study in the context of mechanosensing, as it is the sudden lack of tension that promotes muscle degradation. In other words, the steady-state force applied to the muscle (the homeostatic tone) is suddenly decreased, and the muscle metabolism responds. We find a quick decrease in muscle CSA after a series of drastic parameter changes at the start of our simulations, but it is the kinetics of muscle recovery after atrophy that appear to be more dependent on the type of feedback in the model. In practice, muscle is seen to recover relatively rapidly after very substantial atrophy, with most of the recovery occurring over a 1–2 week period (91). Fig. 13 *a* shows our model predictions with the simplifying assumption that there was no feedback relationship between muscle force per fiber and the CSA in the case of hypertrophy. The curves show a response to a very small decrease of steady-state tone (maximal 0.5% in *black curve)*, and recovery when *f*_st_ returns to its value prescribed by (9) after 120 days. A very slow recovery of homeostatic muscle CSA is found, not in agreement with observations.

However, once we include the feedback, when the force per filament decreases with an increasing CSA, the rate of response becomes much more realistic (see Fig. 13 *b*). Here, a much greater force increase is applied (up to 5% in the *black curve*), and we see both the atrophy onset and the recovery reaching the saturated steady-state values within 60 days. This suggests that a reasonable force feedback scale (with the parameter *μ* ∼0.02 or even higher; see Supporting materials and methods, Section E for detail) is a required feature of our model if quantitative predictions are to be obtained.

An unexpected feature of plots in Fig. 13 *b* is the muscle “overshoot” during the fast recovery after atrophy. It seems likely that the several intrinsic processes have low rate but high sensitivity, resulting in muscle keeping a memory of its previous architecture during atrophy, much like the career-trained athletes whose muscle CSA remains higher than normal after retirement. This would translate into a corresponding increase in the muscle force at smaller muscle CSA.

## Discussion

In this work, we developed a kinetic model combining the intracellular mechanosensor of the second kind, the signaling chain pathway (admittedly one of several), and the ribosomal kinetics of post-transcription synthesis to examine how muscles sense and respond to external load patterns by producing (or degrading) their contractile proteins (41). The important factor of limitations to ATP supply, which affects both the MVC level because of myosin activation and the signaling because of phosphorylation, is included in the background (see Fig. 3; Supporting materials and methods, Section A). The primary marker of morphological response for us is the CSA of an average muscle fiber, which is directly and linearly mapped onto the number of titin molecules per fiber. We suggest that the titin kinase (TK) domain has the right characteristics to play the role of the primary mechanosensor within the muscle cell. By looking at how TK unfolds under force, we found that it acts as a metastable switch by opening rapidly only at high forces but opening and closing slowly within a range of physiological forces. The muscle is known to apply a low-level tensile force and be under a steady-state passive tension at rest, which we compare with the steady-state force predicted by our model. We find that the two forces are of the same magnitude, which suggests that long-term muscle stability is due to a combination of the active muscle tone and the passive muscle load stored in elastic sarcomere proteins, notably titin. We find that small changes in the steady-state force allow the muscle to maintain its size after the end of a resistance training program, and we suggest that this change in steady-state muscle tension might account for some of the “memory” that muscle develops after long-term training (77,78).

Given the switch-like nature of TK, it seems likely that different individuals will have slightly different predispositions toward applying somewhat more or less muscle tone in homeostasis and therefore can maintain muscle mass much more or much less easily. This low-level steady-state tensile force will crucially depend on the number of available myosin heads and on the steady-state ATP concentration in the cell, as well as sarcomere and tendon stiffness.

Our model shows qualitatively reasonable time courses for hypertrophy developing during a regular exercise regime followed by detraining, as well as muscle atrophy followed by recovery. Although it is not explicitly included in this model, long-term changes in muscle filament architecture (slightly increasing the muscle tone with the same CSA), as well as increases in myonuclear number after chronic hypertrophy (increasing the synthesis rates in the model), could cooperate to increase the steady-state muscle CSA. This could then provide a rationale for the observed permanent increase in muscle size after just one bout of resistance training in the past (92). The model uses no free-fitting parameters, as all its constants are independently measurable (indeed, Table 1 gives examples of such measurements). Obviously, there would be a large individual variation between these parameter values, and so applying the quantitative model predictions to an individual is probably optimistic. However, we are excited to develop a software to implement the model and make specific predictions in response to any chosen “exercise regime,” which could be used and adapted to practitioners.

We saw that a successive integration of the initial mechanical signal is necessary for muscle cells to display trophic responses at the right timescales. Indeed, a several-week lag is observed in the increase of structural proteins content after the start of an exercise regime; in our model, this arises because of a lag in ribosome number, as ribosome turnover and synthesis are relatively slow. Research into the sterically hindered diffusion of ribosomes in muscles so far appears to be very much in its infancy, despite its obvious overarching implications in muscle development. This would be an exciting avenue for future research in this area.

To further improve the model, we could include more details about the viscoelastic properties of muscle. Its effects would be twofold: first, the switching kinetics between the titin kinase conformations would change somewhat (see Supporting materials and methods, Section E.2 for more details); secondly, it would allow for a treatment of how muscle fatigue affects the compliance and therefore the mechanosensitivity of the muscle’s structural proteins. We expect the plateauing of the mechanosensitive efficiency at high forces explored in the Supporting materials and methods, Section A.4 (in particular, see Fig. S4) to be even more pronounced. In fact, because the sarcomere structural proteins likely change their mechanosensitive properties at high forces, we expect there to be an optimal force at which the exercise should be carried out. However, this would substantially increase the mathematical complexity of the model, reducing its present intuitive clarity, and make an analytical solution for homeostasis less tractable. This is the next stage of model development that we hope to pursue.

In summary, how intracellular signaling in muscle cells organizes a trophic response is a central question in exercise science and in the study of conditions that affect muscle homeostasis (including development and aging, as well as numerous pathologies). Cells have been shown to use time-integrated mechanical stimuli to initiate signaling cascades in a way that depends on the strength and duration of the signal (i.e., mechanosensitively). This work provides a quantitative analytical rationale for a mechanosensitive mechanism for trophic signaling in muscle and gives an additional piece of evidence that the titin kinase domain is a good candidate for hypertrophic mechanosensing. We expect advances in targeted exercise medicine to be forthcoming, specifically if the exact structure of the mechanosensing complex bound to the TK domain and its downstream signaling cascade are studied in more detail.

## Author contributions

Both authors conceived the idea, carried out different elements of data analysis, and wrote the manuscript.

## Supporting material

Document S1. Supporting materials and methods and Figs. S1–S7

Document S2. Article plus supporting material
