# Supplementary material for: Why exercise builds muscles: titin mechanosensing controls skeletal muscle growth under load
Source: Biophys J. 2021 Aug 10;120(17):3649–63. doi: 10.1016/j.bpj.2021.07.023 (PMC8456289; doi:10.1016/j.bpj.2021.07.023)
Supplement: Document S1. Supporting materials and methods and Figs. S1–S7 [file mmc1.pdf]

Biophysical Journal, Volume *120*

Supplemental information

Why exercise builds muscles: titin mechanosensing controls skeletal muscle growth under load

Neil Ibata and Eugene M. Terentjev

Preamble

In this Supplementary Material, we detail our reasoning behind some of the main assumptions of the model, namely that titin kinase (TK) is a good location for the sarcomeric mechanosensor and that it is under force during muscle contraction, and analyse the literature for the relevant kinetic rate constants and molecular concentrations.

In order to do this, we organise our discussion into five main sections following Fig.3 in the main text. First, in Part A, we consider TK as a mechanosensor: we show why it is a good mechanosensing candidate, and quantitatively justify the kinetic equations and constants in our model. Next, in Part B, we consider how the mechanosensitive signal affects protein synthesis; this relies on physiological constants and a large number of order-of-magnitude estimates. In Part C, we introduce equations for phosphate transfer in the muscle cell; these substantially limit the muscle's ability to signal during exercise but were too complex to include in the main text. In Part D, we analytically solve the combined equations in the steady state. Finally, Part E examines a few asides; in particular, how muscle force per area could depend on the muscle fibre cross-sectional area (CSA), and how our simple model of TK opening detailed in Part A can be improved by including knowledge of how viscoelasticity affects TK kinetics.

PART A: TITIN KINASE AS A MECHANOSENSOR

Part A.1: Discussion of the different possible sarcomeric mechanosensing sites

Mechanosensitive sensors can either be load-bearing molecules (*i.e.* those which are part of the sarcomeric force chain), or auxiliary molecules which measure the deformation in the force chain. The prime candidate molecules are sketched in Fig. S1. MYBPC, which displays a phosphorylation site under tension[1], was for a time suggested as a potential mechanosensor. Karsai et al.[2] used AFM to measured the force-induced unfolding MYBPC (cardiac isoform), and they found that the protein started to unfold under forces of 50pN or more. MYBPC is regularly placed in 7-9 stripes with an axial repeat distance of ca. 43 nm[3,4]. Its stoichiometry was found by[5] to be approximately 37 cardiac MYBPC per thick filament, or roughly three times as many molecules as titin or nebulin. Each MYBPC would then be under lower load individually compared to those other proteins, and require a much greater overall sarcomere force to unfold. In addition, recent work appears to assign MYBPC phosphorylation the role of a regulator for myosin function[6–8], so we disregard MYBPC as a mechanosensor for mechanosensitive signalling.

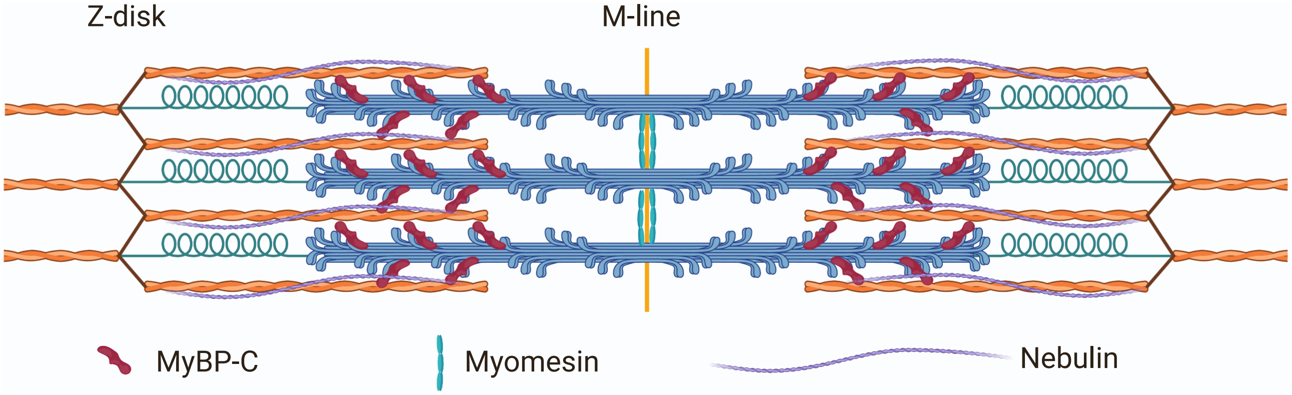

FIG. S1: A sketch of the sarcomere molecules considered in Part A for their possible role in mechanosensing. Nebulin is a poor candidate because of the low compliance of the thin filament[9]; MyBPC is not a tempting choice because it unfolds at high force[2]and is substantially more common than titin[3,4]; myomesin could play a role in transverse strain sensing during sarcomere compression[10–12] but is unlikely to directly sense force in the filaments. Created with BioRender.com

Neighbouring sarcomere units connect at the Z-disks (Z-line) of the sarcomere: regions of extensive α-actinin cross-bridging between neighbouring thin filaments as well as titin Z-repeats, and of telethonin based (Tcap) titin-titin bridging[13,14]. Thin filaments in one sarcomere in a resting muscle interdigitate at the Z-disk[13], aligning with thick filaments in the neighbouring sarcomere (see Fig. 1).

Both the Z-disk and the M-line segment of the thick filament are under tension during muscle action, and it is therefore unsurprising that both have been suggested as possible spots for mechanosensitive signalling[15]. The Z-disk is the location of a large protein plaque (containing more than 40 different different protein types[16]), which interacts

with a large number of cytoplasmic proteins[17], making it a tempting place to search for a specialised mechanosensor molecule. However, because the load is applied differently through the Z-disk in eccentric and concentric muscle movements, its seems unlikely that the same mechanosensitive molecule would be able to act as a load sensor for both types of loading.

The complex formed by the titin Z_1Z_2 repeats, telethonin (Tcap) and muscle LIM protein (MLP) appears to have a mechanosensitive role as a stretch sensor[18–20]. However, due to its location away from the thin filament, this complex seems not to participate in the primary sarcomeric force chain, and thus an unlikely candidate for a mechanosensor for resistance training. It will only be under substantial load when the titin Z-band segment is under tension, which occurs only during eccentric lengthening of titin.

A better candidate for mechanosensing within the I-band (which contains the Z-disk) could be the as-yet not much studied nebulin, which provides structural stability to the thin filament[21]. It has the additional benefit of only being substantially expressed in skeletal muscle (two strands per thin filament) but not in heart muscle, and would thereby immediately handle the 'difficulty' that muscle hypertrophy is not seen in heart muscle. But the compliance of the thin filament is very low[9,22], which means that nebulin does not unfold very much; this suggests that nebulin plays a structural rather than a signalling role. In addition, nebulin, or a similar thin-filament bound molecule, cannot by itself be the only mechanosensor, because hypertrophy was also observed in studies of muscle stretching by overloading, as reviewed by[23]. In experiments where muscles are stretch-overloaded, the thin filament is under minimal tension but the thick filament is stretched beyond the normal PEVK-related elastic response of the muscle[24–26], which would lead to the unfolding under force of other protein domains. Finally, there are twice as many thin filaments as thick filaments, which means that there is overall a 4/3 ratio of nebulin to titin. Provided that both are on the force chain, the force transduced through an individual titin molecule would therefore be larger than for an individual nebulin, making titin a better candidate for mechanosensing than nebulin.

Mechanosensor within the M-band: titin kinase

Due to their arrangement largely perpendicular to the direction of applied force, it seems unlikely that the common myomesin family M-band components[27] play the role of a potential hypertrophic mechanosensor within the sarcomere M-band. They are more likely to be mechanical springs[28], which help keep the sarcomeres aligned during muscle contraction. During muscle contraction, the distance between thick filaments increases (by volume conservation) and they might also play a role in sarcomere strain sensing.

This leaves the titin molecule itself as a possible candidate for a mechanosensor. It is known to possess a kinase domain (titin kinase, TK)[29], which has been suggested as a hotspot for trophic signalling[18]. There are also structural similarities between TK domain and focal adhesion kinase (FAK), which is known to be the principal mechanosensor in the integrin adhesion complexes on the cell periphery[30–32]. Better still, the titin M-band is likely under load both during muscle contraction (which strains the entire thick filament, see Part A.4 below) and eccentric muscle stretching (which strains titin only).

We will hereafter use the working hypothesis that the titin M-band region contains the primary location for the mechanosensor, which signals for hypertrophy to occur (while remembering that other regions of the sarcomere may also contribute to this highly complex process). This hypothesis yields an immediate qualitative explanation for muscle hypertrophy detected after chronic stretching: stretching lengthens the sarcomere, loading titin and titin kinase. A mechanosensor of the second kind allows for intracellular mechanical signals to be integrated over a long period of time, hence why only chronic loading or stretching elicit a trophic response.

Part A.2: Estimate of the maximum force per filament from macroscopic tension

The inter-thick filament spacing of human muscle was found in diffraction experiments to be approximately $d = 46\text{nm}$[33–36]. The lattice of filaments in sarcomere cross-section can be mapped by approximately equilateral triangles, so the area occupied by each filament is simply the area of each triangle of side d, or $A_{\text{filament}} = \frac{\sqrt{3}}{4}d^2 \approx 9.2 \cdot 10^2\text{nm}^2$.

The force-area relation of the maximal force which can be exerted by single muscle fibres (with multiple myocytes) was examined by Krivickas et al.[37] and found to be linear as a function of area in untrained individuals, with a higher maximal force generated in younger fibres for the same cross-sectional area. For young muscle, they found a proportionality constant $K_{\text{avg}} \approx 0.17\text{pN}\,\text{nm}^2$. For some fibres more force was produced: $K_{\max} \approx 0.3\text{pN}\,\text{nm}^2$, while for others (especially older fibres), smaller values of $K_{\min} \approx 0.08\text{pN}\,\text{nm}^2$ were measured.

This means that we should expect a maximum force per filament of the order of $f_{\max,\text{avg}} = K_{\text{avg}}A_{\text{filament}} \approx 320\text{pN}$ (range $150 - 500\text{pN}$) in untrained individuals. We expect that long-term resistance training should shift this figure towards the upper end of the range, for instance by recruiting more myosin heads under tension (*e.g.* by increased

neuronal activation[38] or stretch activation[39]). The net result is that the upper physiological limit for the maximum force per filament is probably of the order of $\lesssim 500$pN. This value is consistent with the maximum contraction force in the data obtained by Ma et al.[40] (see Fig. S2 below). There are six titin molecules per thick filament on either side of the sarcomeric M-band, so these values must be divided by 6 to obtain the maximum force per titin, and therefore per TK mechanosensor.

Part A.3: Estimate of the maximum force per filament from the action of myosin heads

In the sliding filament hypothesis, myosin filaments 'walk' along the thin F-actin filaments with a characteristic force-velocity dependence. The fraction of the myocyte which is occupied by myofibrils depends on the muscle type, and is typically in the range of $70-80\%$[41]. The stall force of myosin-II, which is appropriate during isometric muscle contraction, is found to be ca. 5.7pN[42]. The presence of too many active myosin heads per filament was found to lead to a buckling of actin and so a maximum number of myosin heads could be simultaneously engaged on a thick filament, in a way that was found to be dependent on ATP and presumably also depends on postranslational modifications of nebulin and other structural molecules. Various studies have found this number to be in the range of 90 ± 25[43–46] active myosin heads per filament. Myosin-II has a low duty ratio, that is, the proportion of time during which it is bound to actin is low. The duty ratio is found to be ATP dependent[47,48] and to be much higher during isometric contractions (up to 0.22 in *R. esculenta*) compared with an unloaded sarcomere (ca. 0.05)[49–52]. The total (bound and unbound) number of myosin heads per filament was found to be approximately 588[53] (294 within each half-sarcomere), so the maximum number of heads per filament while working at the maximum duty ratio can be estimated to be ca. 120.

Assuming that myosin is working at its maximum duty ratio in optimal ATP conditions (which we assume corresponds to the maximum isometric load in a tetanic contraction), the maximum force per filament would be $f_{\text{filament}} \approx 120 \cdot 5.7 \approx 700$pN, which matches well with the value obtained from diffraction patterns in frogs by[54], who found $f_{\text{filament}} \approx 297 \pm 40$pN per **actin filament**. The force per thick filament would then be double the force measured in the thin filament.

This is rather more than our qualitative estimate of the force per filament in well-trained individuals. This discrepancy could arise from a combination of effects: the muscle cannot fully coordinate the maximal activation of several fibres at the same time, voluntary contraction is less strong than tetanic contraction (only some 42% of the total myosin heads bind to the actin filaments[55]), and the maximal force only lasts a very short period of time. Mechanosensing of the second kind, as applied to TK, requires the sarcomere force to last a substantial period of time (of the order of seconds) for the mechanosensitive signalling complex to form, so it seems unlikely that a peak per filament force occurring very briefly somewhere in the muscle would substantially change the overall kinetics of titin kinase mechanosensitive signalling.

Because of this, we choose to use the maximum filament force produced by voluntary contractions extrapolated from macroscopic force observations to indicate the typical maximum thick filament load.

The maximum force per titin ultimately depends on the relative force-extension curves of titin and myosin (see discussion in Part A.4 below). There are 6 titin molecules per half-sarcomere, so the upper bound on the force on titin could be of the order of $\approx 30-80$pN for untrained individuals ($\lesssim 80$pN after training); in reality, the force on titin is much lower because myosin appears to be the main load-bearing element during muscle contraction (see Part A.4 below).

Part A.4: Estimates of titin and myosin thick filament extension under load

Titin indirectly connects to the thick filament in the A-band by interacting with Myosin Binding Protein C cross-bridges[56]. Muscle myosin spends much of its time in an inactive and relaxed conformation where it is detached from the thin actin filament. It is sensible to assume therefore that muscle titin largely interacts with the thick filament when it is relaxed. So, if the thick filament stretches, then the part of titin held in place between the cross-bridges, namely its M-band, would stretch in concert with the thick filament by at least the amount that the myosin backbone stretches. When the thick filament extends during muscle contraction, titin is placed under at least some tension and extended. The essential question is to determine how much titin can extend; in order to do that, we must compare titin and thick filament force-extension curves.

The force on titin kinase when the myosin thick filament is loaded depends fundamentally on the force-extension curves of titin and myosin. We assume, for the sake of simplicity, that M-band titin and the thick filament extend in tandem. If titin does not coil around the thick filament (see Fig. 2 in the main text), then this assumption should be quite good.

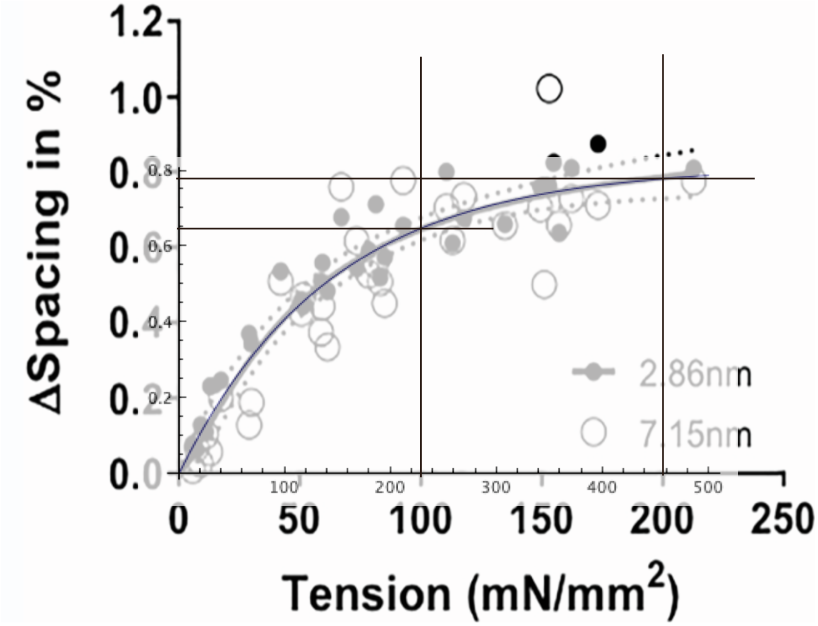

FIG. S2: Data for thick filament extensibility obtained by Ma et al.[40], overlayed by our fit in Eqn 1. To convert from whole muscle tension (in mN/mm^2, we scaled the number by the CSA area occupied by a single filament, and divided by 0.8 (an estimate of the myofibrillar density in a typical skeletal myocyte). See Fig. 1 in the main text for more details.

Ma et al.[40] found thick filament force-extension curves, which suggest that the thick filament was much more compliant (up to $\approx 1\%$ length change) than was originally found in diffraction experiments by Wakabayashi et al.[22]. This apparently paradoxical situation was resolved by Reconditi et al.[57], who showed that the extensibility of the thick filament arises from a 0.3% elastic when the sarcomere is under the maximum load that it can generate, as well as a much larger structural change when the myosins are initially activated. Together, these two values can account for the force-extension curves found by Ma et al.[40]. By avoiding questions of stiffness and compliance per se, and only considering extensibility, we can avoid the thorny details of this problem.

Their data (see Fig. S2) is fit to a good approximation by a decaying exponential fit (f in pN):

$$\frac{\Delta L}{L} = \frac{6.5}{8}(1 - e^{-0.007f}) \tag{1}$$

The thick filament length is $\approx$ 800nm for the half sarcomere, so the thick filament force-extension relation is approximately:

$$z = 6.5(1 - e^{-0.007f}) \tag{2}$$

where the extension z is expressed in nm.

We do not know of a full study of M-band titin stretching under force, so we assume that TK is the most compliant segment of M-band titin and accounts for most of its stretch. AFM data obtained by Puchner et al.[58] show that TK stiffness increases rapidly until it unfolds under force (see Fig. S6 below). Qualitatively, their data suggests that the WLC model is a good model for the extension of TK under increasing load prior to any domain unfolding. The following parameters are a good fit for the curve showing the extension of the closed state of TK in Fig. S6 (see below; data from Puchner et al.[58]):

$$f = \alpha_1\Big(\frac{1}{33.1}(z + 19.6) + \frac{1}{4(1 - \frac{1}{33.1}(z + 19.6))^2} - \frac{1}{4}\Big) \tag{3}$$

where z is the extension of M-band titin in nm and $\alpha_1 = 5 * 10^{-12}$ is a scaling factor. Evaluating this formula yields an average extension of $z_1 = 4.7$nm at $f_1 = 20$pN and $z_2 = 7.4$nm at $f_1 = 40$pN.

These two empirical and graphical fits can be combined to express force per titin in terms of force per myosin. This approach has an obvious limitation, because the TK force-extension traces obtained by Puchner et al.[58] are biased

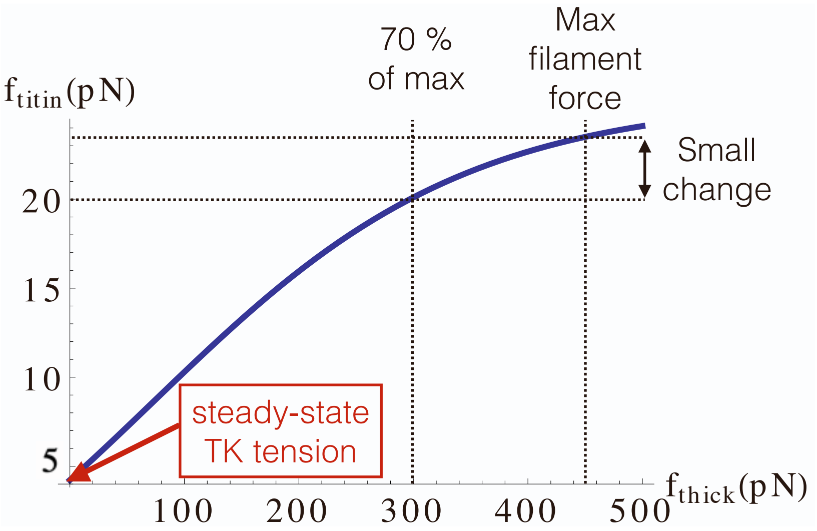

FIG. S3: Adjusted force per titin as a function of thick filament force, assuming a constant 5pN overestimate of the titin force in AFM data due to excessively fast pulling. Note how titin force plateaus at high thick filament forces. We suggest (see also Fig. S4) that this effect accounts to some extent for exercise at the maximum voluntary force not being more efficient than exercise at ≈ 70% of that force.

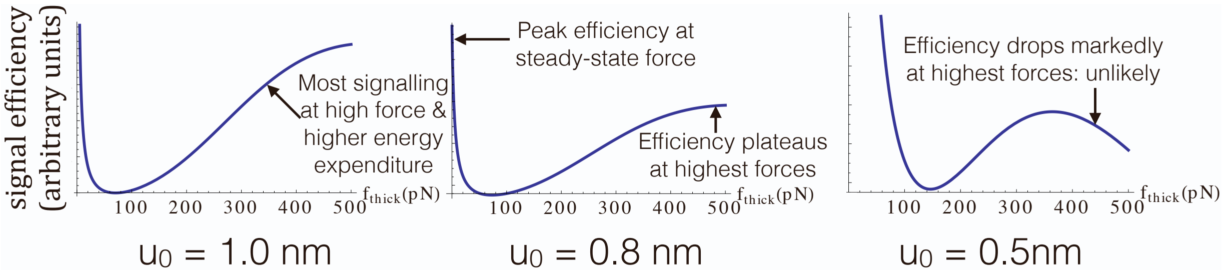

FIG. S4: Plot of mechanosensitive signalling at a constant energy expenditure – or signaling efficiency (arbitrary units). Note that both the steady-state force ≈ 5pN (active muscle tone in the thick filament) and high-load ($\gtrsim$ 70% of the maximum voluntary contraction force) are favoured regimes. The opening distance of TK, which has been estimated in MD studies of TK opening[59] is an important predictor of whether or not mechanosensitive signaling efficiency continues to increase, plateaus or presumably begins to decrease (see right-most panel for a small value of u_0 at the highest forces. Our simulations used $u_0 = 1.0$nm to match up with the predictions from MD simulations for the TK opening distance[59].

by a non-negligible pulling speed; this will make estimates of force per titin using this method likely several pN too high. If this overestimate is of the order of 5pN, the relationship between TK force and myosin force is consistent with our estimate of titin steady-state force $\approx 4pN$ and informs our estimate of titin force at 70% of the 1 repetition maximum ≈ 20pN (see main text). For the sake of simplicity, we assume that the force overestimate is a constant 5pN throughout the range of TK forces, and find the trace in Fig. S3.

In Fig. S3, titin force grows more slowly as the thick filament force increases beyond ≈ 70% of the maximum voluntary force. We suggest that this cutoff might help explain why exercise at the maximum voluntary contraction force is not substantially better at inducing muscle hypertrophy than exercise at ≈ 70% of that force. If we assume, for the sake of simplicity, that mechanosensitive signaling is approximately proportional to TK opening rates and exercise duration, we see from Fig. 7 in the main text that TK opening rates depend exponentially on force, whereas exercise duration for an equal amount of work decreases linearly with myosin force. By multiplying $e^{\frac{f_{\rm titin} u_0}{k_B T}} \times \frac{1}{f_{\rm thick}}$, we obtain a mechanosensitive signaling efficiency in arbitrary units, which we plot for different values of u_0 in Fig. S4.

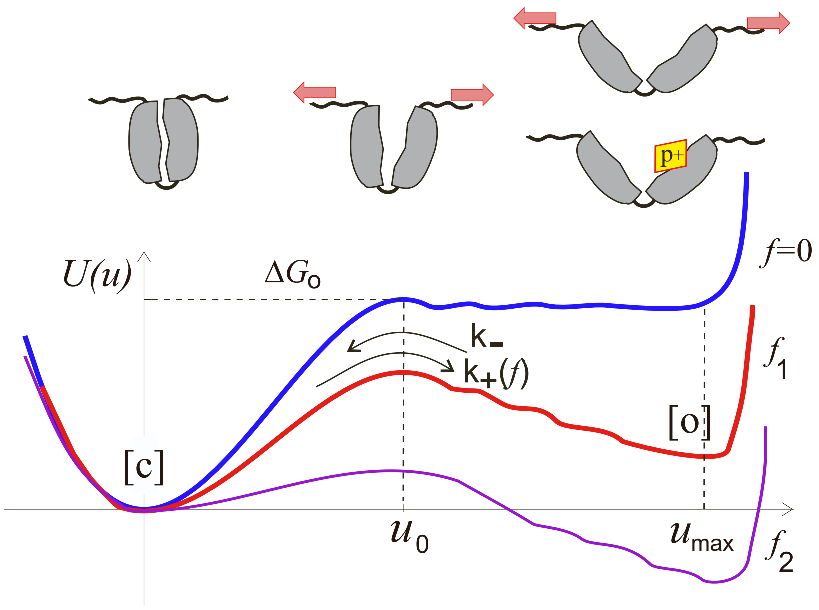

FIG. S5: Sketch of the tilting of the TK energy landscape in favour of the open conformation as the force on the molecule increases. At zero force, there is an energy gap ΔG_0 between the closed [c] and the open [o] conformation. The energy step occurs between the contour length corresponding to the initial conformation (zeroed for simplicity) and a contour length u_0. As the force on the molecule increases, the open state becomes metastable, with a local minimum at a position $u_{\rm max}$. Finally, once the force increases past a critical value (see Fig. 6 in the main text), the open conformation is favoured in the steady-state. The activation energies for the opening and closing reactions (see Eqns. 10 and 11) are the height differences between the step at u_0 and the closed and open states at the origin and $u_{\rm max}$ respectively. The sketch above illustrates the domain opening, and the interpretation of the distances u_0 and $u_{\rm max}$.

Part A.5: Analysis of the AFM data for titin kinase opening and phosphorylation rates

Here, we will consider how TK time-integrates M-band tension inputs and how it uses a metastable mechanically denatured conformation to store this information. FAK (see main text) and TK behave rather differently when they are stretched: as a protein domain rather than a whole protein, TK responds not only to the substrate's viscoelastic properties but also to those of the other segments of the molecule (e.g. the PEVK domain of titin has lower stiffness than TK and will unfold first during fast eccentric exercise, accounting for much of the muscle's elastic response[24,60–62]; we will avoid this complication in this work).

The FAK opening and closing kinetics under force are a good starting point from which to consider mechanically-induced conformational changes in TK. We suggest that the open (which supports ATP-binding) and closed (where phosphorylation is impossible) states of TK can be identified in AFM data of force-induced titin unfolding measured by[58]. The energy barrier between the two conformations can be deduced from the force-extension data and determines the opening and closing kinetics of the domain.

In AFM experiments, the measured force increases until it overcomes the energy barrier between two conformations, at which point the molecule suddenly unfolds and the force drops to a low value. This yield a characteristic saw-tooth pattern, as found e.g. in classical traces of Ig domain unfolding[63]. When the contour length of the protein (relative to the AFM cantilever) increases from x_1 to x_2 under force, physical work $W_{\rm extend} = \int_{x_1}^{x_2} f(u) du$ is done, where $f(u)$ is the force trace in the AFM experiment. In other words, the energy barriers between the states in AFM experiments can be obtained by integrating the AFM traces between two neighbouring states.

With this in mind, we examined the TK domain AFM traces measured by Puchner et al.[58] in some detail in Fig. S6. The first 'peak' in the force-length trace can be discarded as it represents the unfolding of the linker between the TK domain and the AFM tip, which means that the actual unfolding of the TK domain is analysed after further extension. It seems likely that the second peak – which in Puchner et al.[58] is labelled 2 or A in their Figs. 2 and 3 – corresponds to the transition from the native 'closed' to the 'open' conformation in which the ATP-binding site is exposed. This connection is reinforced by the observation of a new peak, labeled respectively 2* and D in their Figs. 2 and 3, which appears in media containing high concentrations of ATP (see Fig.2d of[58]). Since the transition from

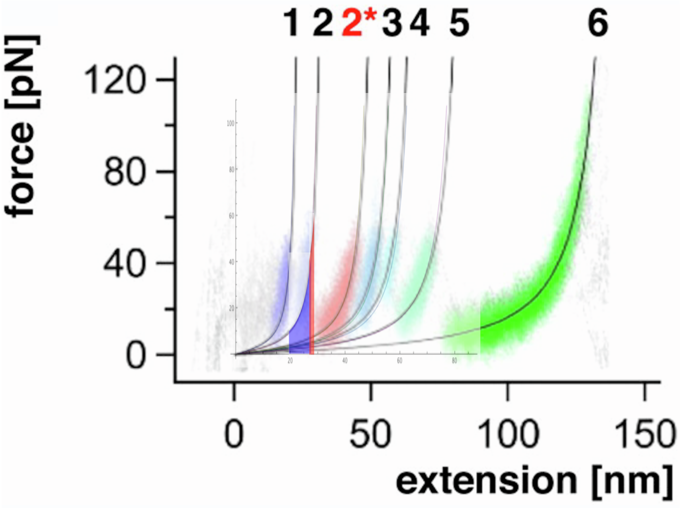

FIG. S6: Fit to the force-extension curves using the worm-like chain WLC model ($f \propto z + \frac{1}{4(1-z)^2} - \frac{1}{4}$)[64], where z is scaled so that the molecule is maximally unfolded when $z = 1$. By scaling the WLC formula to the peaks found by Puchner et al.[58] as $f = \alpha_1 \left(\alpha_2 z + \frac{1}{4(1-\alpha_2 z)^2} - \frac{1}{4} \right)$ with the proportionality constants: $\alpha_1 = 5 \cdot 10^{-12}$pN and $\alpha_2 = 1/l_c$ where l_c is the contour length of the unfolding peak of the molecule in SI units, we find a very similar fit to the one shown in their Figs. 2B and 2C, where the extension z and the force f are now expressed in SI units.

the open to the phosphorylated state is assumed to be force-independent, it is likely that the new peak corresponds to a transition to another, further extended state. The transition from the open to the phosphorylated states then necessarily occurs before this point, which gives their peak labelled 2 as the sole candidate for the transition between the closed and open TK conformations.

Estimate of the opening energy ΔG_0: The opening energy of the TK domain can then be deduced from the energy under the curve between the 1st peak (corresponding to the unfolding of the AFM linker) and the 2nd peak (when the TK domain opens). In order to account for energy dissipation during pulling, we must subtract the extra area above the WLC fit to the right of each of the force peaks (see Biswas et al.[65] for a graphical explanation). The resulting area may still reflect some elastic energy stored in the other parts of the molecule. However, these open at much higher extensions (curve 6) and only store a small amount of energy at low extensions corresponding to the domain opening. In fact, some of the peaks found by Puchner et al.[58] require TK to be open first (specifically 2* because the ATP molecule can only bind to open TK, and possibly also peaks 3,4,5, which correspond to more unfolded conformations of TK, see MD simulations in Puchner et al.[58] Fig. 3). Because of this, we consider the elastic energy stored during the opening of the initial peak to be restored when the molecule opens and transitions between the closed and the open states of TK. The mean unfolding force is uncertain and a proper estimate would require a more detailed analysis of the stochasticity of TK opening process; we graphically find it to be between 40 and 60pN. Integrating the WLC formula between the corresponding extensions $z_1 \approx 19.6$nm and $z_2 = 27.0$nm for the lower unfolding force estimate, or $z_2 = 28.2$nm for the higher unfolding force estimate, we would estimate the unfolding energy to be in the range ca. $30k_BT < \Delta G_0 < 50k_BT$ (lower estimate from the blue area under the curve, higher value from the red area under the curve). Since we believe this to be a slight overestimate, and choose the bottom of the resulting range as our most likely value of $\Delta G_0 \approx 30k_BT$ (see Fig.4 in the main text). Note also that Puchner et al.[58] use a slow pulling rate of 300nm/s for the 25 nm TK domain, which approaches the physiological rate of sarcomere contraction in real muscle. This is to be contrasted with out-of-equilibrium molecular dynamics (MD) simulations at much higher pulling rates (which Puchner et al.[58] also do). MD simulations were used, in conjunction with models for how globular molecular domains can open[66], to find that the free energy difference between the closed and open states of the similar focal adhesion kinase was $\approx 28.5k_BT$[67]. This value is in the same ballpark as the $30k_BT$ value that we estimate here.

The analysis in Fig. S6 suggests that the TK opening energy is in the range $25k_BT < \Delta G_0 < 45k_BT$.We will test our model across this range of possible values. Molecular dynamics simulations shown by Puchner et al.[58] in their Figure 3 suggest that the maximum unfolding distance of the relevant TK domain is $u_{\max} \approx 28$nm, which we will use as an important model parameter. Note that the value for $u_{\max}$ could be a little smaller in the presence of ATP, where another energy peak develops and skews the energy landscape.

Modelling the kinetics of conformational changes of the TK mechanosensor

In the absence of any signalling, the transitions from closed to open to phosphorylated TK domain conformations were found in the main text to be simply described by the kinetic equations:

$$\frac{dn_{\rm c}}{dt} = -k_+ n_{\rm c} + k_- n_{\rm o} + k_- n_{\rm p} \tag{4}$$

$$\frac{dn_{\rm o}}{dt} = k_+ n_{\rm c} - k_- n_{\rm o} - k_p n_{\rm o} {\rm ATP}_{\rm free} + k_r n_{\rm p} \tag{5}$$

$$\frac{dn_{\rm p}}{dt} = k_p n_{\rm o} {\rm ATP}_{\rm free} - k_r n_{\rm p} - k_- n_{\rm p} \tag{6}$$

$$n_{\rm c} + n_{\rm o} + n_{\rm p} = n_{\rm total} \qquad \text{(constraint)} \tag{7}$$

where the last equation encodes the (near) constant concentration of titin per unit volume. Titin conformational changes are fast (less than a second) and the total concentration of total (free + bound) ATP can be assumed to be constant over such very short times.

These equations are shown in Part A.6 to adequately reproduce the phosphorylation kinetics of TK, thus providing an a posteriori justification for their use.

Rate constants for titin mechanosensor conformational changes

In the next section, we discuss how experiments by[58] enable us to determine order of magnitude values for the TK phosphorylation and de-phosphorylation constants k_p and k_r. We will see that $\{k_r = 6s^{-1}, k_p[\text{ATP}] = 35s^{-1}\}$ are a good fit to data obtained by Puchner et al.[58] and can be used as ballpark values for de-phosphorylation and phosphorylation rates.

The rates of TK opening and closing under force are more complex. To our knowledge, no experiments have attempted to measure these rates directly for TK, and so we must rely on studies of other molecules as well as theoretical tools to examine them. In the most basic model of conformational change between two molecular states, an extension of Arrhenius kinetics gives us some qualitative insight into the transition rates between the two states:

$$k_+ = w_0 \exp\left(\frac{-E_+^\ddagger}{k_B T}\right) \quad \text{(opening)}, \qquad k_- = w_0 \exp\left(\frac{-E_-^\ddagger}{k_B T}\right) \quad \text{(closing)} \tag{8}$$

where $E_+^\ddagger$ and $E_-^\ddagger$ are the activation energies of the forward and backward reactions respectively (see Fig. S5 for a graphical illustration). w_0 is not the number of collisions between molecules as in classical Arrhenius theory, but the number of attempts at crossing the barrier. This value is well known in polymer theory as the inverse of the Rouse time corresponding to relaxation time of the $p = 1$ Rouse mode within the polymer:

$$\tau_{p=1} = \frac{N^2 b^2 \gamma_{TK}}{3\pi^2 k_B T p^2} = \frac{N^2 b^2 \gamma_{TK}}{3\pi^2 k_B T} \tag{9}$$

where N is the number of amino acids within the protein segment, γ_{TK} is the dissipative friction coefficient internal to the TK domain, b is the size of each amino acid and k_B is the Boltzmann constant. If the internal friction coefficient of TK is similar to that of FAK[32], we estimate $\gamma_{TK} \approx 10^{-9}\text{kg s}^{-1}$.

The length of TK at the energy barrier between the closed and open conformations is reported by[58] as: $l = 9.1$nm. If we assume that vibrations in the polymer which can induce conformational change occur largely within the free section of the molecule, then the number of amino acids within the molecule which contribute to the above calculation is much less than the total number of amino acids within the molecule and so $Nb \approx 9.1$nm, giving an estimate of the Rouse time as $\tau_{p=1} \approx 6.5 \cdot 10^{-7}$s. As an order of magnitude value, we shall take the number of attempts to be $w_0 \approx \frac{1}{\tau_{p=1}} \approx 10^6\text{s}^{-1}$.

For the sake of simplicity, we will assume that the number of attempts w_0 is similar for the opening and closing rates. (Note that they tend to the same value if the molecule adiabatically tends towards the transition between the closed and open states, while they will be somewhat different if the molecule suddenly jumps from one conformation to another. We we discard this complication.)

The activation energy corresponds to the energy between the trough in the energy landscape found at the stretched length at which a particular molecular conformation is most stable and the peak in the energy landscape between two neighbouring conformations. Placing titin under tension tilts the energy landscape as a function of TK stretched length u by an amount $\Delta E(u) = -\int_{u_{\rm init}}^{u} f_{\rm applied} du'$. We assume that the peaks and thoughs in the landscape are sufficiently well-defined that pulling on the molecule does not change the location of the peaks and troughs, but only their relative heights.

The change in activation energy of the opening reaction is easier: the height of the peak in the absence of applied force is simply the integral of the force-trace of the first peak in the AFM data which we take to be $30k_BT < \Delta G_0 < 50k_BT$. Its energy is modulated by an amount $\Delta E(u_0) = -f_{\text{applied}}u_0$, where u_0 is the position of the transition between the open and closed conformations. Work on polymer unfolding suggested that the opening distance for two amino acids, *e.g.* in titin Ig domains, is of the order of $u_{0,\text{Ig}} \approx 0.3$.[63] However, MD simulations by[59] suggest that titin kinase domain opening requires two β-sheets to separate, which takes place over a larger scale of $u_0 \approx 1$nm. The activation energy of the opening reaction is therefore:

$$E_+^\ddagger = \Delta G_0 - f_{\text{applied}}u_0 \quad . \tag{10}$$

When calculating the change in activation energy of the closing reaction, we assume that there is no barrier in the return process in the absence of force; that is to say that the open state is only stabilised by force. The activation energy of this process is then

$$E_-^\ddagger = f_{\text{applied}}(u_{\text{max}} - u_0) \quad , \tag{11}$$

where u_{max} is the contour length of the most stable 'open' TK conformation. This is at the very most the contour length of the peak labelled 3 by Puchner et al.[58] in their Figure 2. Because of this, $u_{\text{max}} = 28$nm seems to be an upper bound for this value.

In reality, the viscoelastic properties of titin and its surrounding media will have an impact on these rates. We touch on this complication in Part E.2 below.

Part A.6: Phosphorylation and de-phosphorylation rates of the open TK domain

Puchner et al.[58] show in their Figs. 2D and 2E that a new peak appears in their AFM measurements in an ATP- and time-dependent manner. The presence of this peak strongly suggests that TK does indeed allow for its open conformation to be phosphorylated. At the start of the AFM experiment, the TK domain is closed, so the peak induced by the phosphorylation of the domain is absent.

A simplified subset of Eqns. 1,2,3 and 4 in the main text allows us to focus on the transition between the open and the phosphorylated state once the closed TK domain has opened. AFM experiments allow for no doubt that the transition has occurred, as the force trace identifies the transition from one state to the next. In the absence of any signalling or closed TK domains, we have:

$$\frac{dn_\text{c}}{dt} = 0 \qquad n_\text{p}(0) = 0$$

$$\frac{dn_\text{o}(t)}{dt} = -k_p[\text{ATP}]n_\text{o} + k_r n_\text{p} \tag{12}$$

$$\frac{dn_\text{p}(t)}{dt} = +k_p[\text{ATP}]n_\text{o} - k_r n_\text{p} \tag{13}$$

Naturally, a single TK domain will not appreciably change the ATP concentration of the solution, so [ATP] may also be considered constant.

These equations are trivial to solve, and give us the probability as a function of t and [ATP] as:

$$P_{\text{Phosphorylated}}(t, \text{ATP}) = \frac{\text{ATP } k_p\big(1 - e^{-(k_r + \text{ATP } k_p)t}\big)}{k_r + \text{ATP } k_p} \tag{14}$$

Its value quickly tends to its asymptote as t becomes large. This can be read graphically from their figure 2E as:

$$0.75 < P_{\text{Phosphorylated}}(t \to \infty, \text{ATP}) = (1 + \tfrac{k_r}{\text{ATP } k_p})^{-1} < 0.9 \tag{15}$$

$$0.75^{-1} - 1 > \frac{k_r}{\text{ATP } k_p} > 0.9^{-1} - 1$$

$$\frac{1}{3} > \frac{k_r}{\text{ATP } k_p} > \tfrac{1}{9} \tag{16}$$

These bounds are very generous to accommodate the substantial error margin within their data. For simplicity's sake, we shall use a middling value:

$$\text{ATP } k_p \approx 5k_r \quad . \tag{17}$$

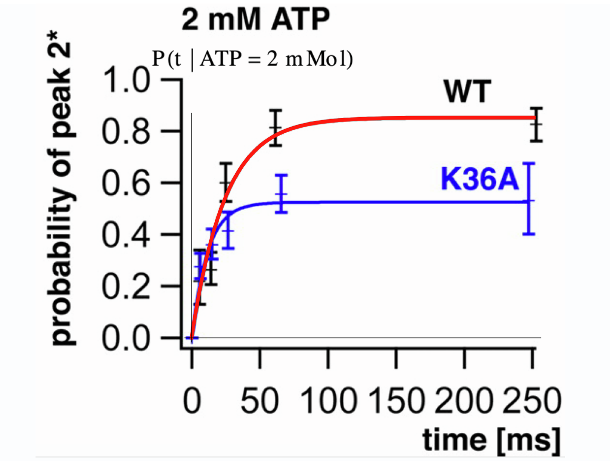

FIG. S7: Plot of the phosphorylation probability of open TK given an ATP concentration of 2mM and $\{k_r = 6s^{-1}, k_p[\text{ATP}] = 35s^{-1}\}$ (red line), which provides a very good of the wild-type phosphorylation probability found by Puchner et al.[58] in their Figure 2.E (black trace and error bars below). Note that many other traces with $\{k_r, k_p\}$ values which differ by a factor of 2 or even 3 from these would also lie within the substantial error margin of their experiments, so we take the above values of k_r and k_p simply as indicative, order of magnitude values which need further measurements to be more accurately determined.

We obtained a perfect fit for Fig. 2E in Puchner et al.[58] with $\{k_r = 6s^{-1}, k_p[\text{ATP}] = 35s^{-1}\}$ (see Fig. S7). Fig. 2D was much harder to fit, presumably due to the tiny number of measurements and large error bars. We will use the values obtained for the fit to Fig. 2E in[58] in the remainder of this work. Note also that the values obtained for the fit to Fig. 2E fit the results from their Fig. 2D as well.

Part A.5: Evidence for a mechanosensing complex bound to TK

How exactly does the phosphorylation of titin kinase lead to signalling? Lange et al.[68] found a set of complementary sequences which suggested a 4-fold binding pattern: open and phosphorylated TK is susceptible to binding by the zinc-finger protein nbr1, which can in turn bind ubiquitin-associated p62/SQSTM1. This has a binding site for muscle-specific RING-B-box E3 ligase MuRF-2, which can bind to the transactivation domain of the serum response transcription factor (SRF). nbr1, p62 and MuRF-2 can all bind as multimers (they suggest as a dimer).[69] They observe that MuRF only translocates to the nucleus during periods of mechanical inactivity, indicating that the signalling complex might be stably localised to the TK domain for some time after exercise.

NB: The following reasoning and approximations carry higher uncertainty relative to the other parts of the text. nbr1, p62 and MuRF have not been studied as well as other muscle proteins, and their concentrations can only be inferred approximately from RNAseq and protein degradation data. Regardless of the exact composition of the signalling complex and of the abundance/binding kinetics of its constituent parts, the goal of this section is simply to obtain a multiplicative scaling factor α between the maximum possible rate of signalling complex creation k_s[70] and its actual aggregation rate k_s'.

The binding of nbr1 to TK can be described by the Hill equation[71]:

$$\left(\frac{dn_s}{dt}\right)_{\text{initiation}} = \frac{k_s \cdot n_{\text{p}}}{1 + (n_{\text{nbr1}}/(\sigma n_{\text{titin}}))^{-m_{\text{nbr1}}}} \tag{18}$$

where n_p is the fraction TK domains which are phosphorylated, k_s is the maximum rate of nbr1 binding to phosphorylated TK, n_s is the fraction of TK receptors which is bound to nbr1 (expressed in number per titin), σ is the number of nbr1 per titin at which half of the TK binding sites are occupied, and m_{nbr1} is the Hill coefficient (which accounts for cooperativity in the nbr1 binding kinetics and can take on non-integer values).

If the binding rates of each of these proteins are much higher than their dissociation rates (which seems likely if a signalling complex is indeed to form while TK is transiently open during and after resistance exercise), the concentration of nbr1 at which the half of the total TK domains support a signalling complex (or at which signalling is at half it maximum value when TK is open) will be of the order of 1 molecule per titin. In other words, the constant σ is of order 1.

p62 binds to nbr1 and MuRF binds to p62 according different Hill aggregation kinetics. However, if the complex has to activate signal in order to disassemble (*e.g.* by SRF activation), then it will have to assemble fully once its assembly has been initiated. In addition, if the signalling (or disassembly) rate of the complex is slow, perhaps because of a lack of available SRF, then the initiation rate of the complex serves as a good proxy for the aggregation rate of the complex. TK bound to nbr1 then serves as a proxy for TK bound to the entire signalling complex, and we need only introduce one more state n_s representing TK bound to the signalling complex. We approximate its aggregation kinetics as:

$$\left(\frac{dn_s}{dt}\right)_{\text{aggregation}} \approx \frac{k_s \cdot n_{\text{p}}}{1 + (n_{\text{nbr1}}/(\sigma n_{\text{titin}}))^{-m_{\text{nbr1}}}} \tag{19}$$

The tricky part is to find qualitative values for the number of nbr1 molecules per titin. Until the number of nbr1 in a muscle cell is directly measured, we must rely on estimates of degradation and synthesis rates to estimate it.

Not all of these molecules are equally expressed, nor do they have the same degradation rates. Degradation rates for nbr1 and p62 have been measured in human embryonic kidney cells[72] by fluorescent flow cytometry. They found a marked difference in degradation rates between nutrient-rich and starved cells[73]. The muscle will typically be in nutrient rich conditions apart from the duration of the exercise bout, so we will take the degradation rates of nbr1 obtained in those conditions to be representative, giving $t_{1/2\text{nbr1}} \approx 10$ hours, and $t_{1/2\text{p62}} \approx 14$ hours. Degradation rates of MuRF family proteins are less well established, but experiments by[74] indicate that it is stable for substantially more than 4 hours. Out of simplicity, let us assume that its half-life is of the same order as the other two molecules in the complex.

Finally, titin degradation rates are surprisingly fast for a sarcomere structural protein, with a measured half-life of $t_{1/2\text{titin}} \approx 3$ days[75] (note that the sarcomere structural proteins are actually replaced at a faster rate, so individual molecules can be integrated into several different sarcomeres over their lifetime[76]).

Absolute synthesis rates of these proteins are difficult to ascertain, but we can get acceptable order-of-magnitude estimates of their relative synthesis rates compared with known proteins from RNAseq mRNA expression data. This comparison ignores any potential translational regulation of the synthesis of these proteins, but it is better than nothing at all. A slightly underpowered RNAseq atlas of muscle cell expression in mice and rats was made by[77]. Approximate readings from their dataset indicate that mRNA abundance between the titin/nbr1/p62/murf/srf species are in the approximate ratio: 3500/40/190/70/10 (skeletal) and 1260/50/150/90/20 (heart).

Assuming for simplicity that all of these proteins undergo the same degree of post-transcriptional regulation, we estimate the abundance of titin/nbr1/p62/murf/srf proteins including knowledge about their synthesis and degradation rates as follows (by multiplying the mRNA synthesis rates by the protein degradation times and dividing by 100): 2500/4/27/7/1 (skeletal) and 900/5/21/9/2 (heart). Obviously, each of these values has a substantial uncertainty which is hard to quantity due to the small sample size of the RNAseq data (6 cells per muscle type).

We can now estimate the proportionality constant between the maximum possible rate of signaling complex aggregation and its actual rate:

$$k'_s = \frac{1}{1 + (n_{\text{nbr1}}/(\sigma n_{\text{titin}}))^{-m_{\text{nbr1}}}} k_s \approx (n_{\text{nbr1}}/(\sigma n_{\text{titin}}))^{m_{\text{nbr1}}}\, k_s \tag{20}$$

where $\sigma \approx 1$ is the nbr1 concentration per titin at half-occupation (assuming strong binding). The cooperativity constant m_{nbr1}[78,79] is slightly less than 2 ($m_{\text{nbr1}} = 2$ would represent two nbr1 molecules binding at the same time; any deviation from this ideal dimer binding would decrease the cooperativity constant somewhat). Combining these estimates, we find that the signaling rate is decreased relative to its maximum possible rate by a factor of $10^{-4} \lesssim \frac{k'_s}{k_s} \lesssim 10^{-5}$ because the density of nbr1 is quite low.

The fastest possible rate at which the mechanosensing complex can initiate its assembly, k_s, is unknown, and depends on the rate of binding of nbr1 to titin. Lange et al.[68] suggested that titin binds to the PB1 domain of nbr1. This domain is notable because it also switches between a folded and unfolded (presumably active) conformation. The folding and unfolding kinetics of this domain were determined by[80], who found that the opening rate of the domain was quite slow, at $0.032 \pm 0.007\text{s}^{-1}$. In the hypothetical scenario where the concentration of nbr1 was much larger than when half of the TK domains are populated by signalling complexes, the maximum rate of mechanosensing complex formation would not arise from diffusive constraints, but would be limited by the opening rate of the PB1 domain. This would in turn fix the scaling constant in the Hill equation to be $k_s \approx 0.032 \pm 0.007\text{s}^{-1}$.

If we take into account the number of nbr1 molecules which are bound to TK, the number of nbr1 molecules could potentially vary with time as follows:

$$\frac{dn_{\text{nbr1}}}{dt} = \left(\frac{s_{\text{initial}}}{s}\right)^{\gamma_{\text{nbr1}}} k_{s,\text{nbr1}} n_{\text{rRNA}} - k_{d,\text{nbr1}} n_{\text{nbr1}} - \beta \frac{k_s(n_{\text{p}})}{1 + (n_{\text{nbr1}}/(\sigma n_{\text{titin}}))^{-m_{\text{nbr1}}}} \tag{21}$$

where $k_{s,\text{nbr1}}$ and $k_{d,\text{nbr1}}$ are nbr1 synthesis and degradation rates and $\gamma_{\text{nbr1}} = 2$ accounts for nbr1 binding as a dimer.

We could include the dynamics of nbr1 in our equations, but such an attempt would be complicated by the documented decrease of lysosomal activity during hypertrophy. As we are not aware of any studies specifically examining the concentration of nbr1 during muscle atrophy or hypertrophy, we make the rather bold assumption that the rate of signalling complex aggregation is constant:

$$k_s = \frac{\tilde{k_s}}{1 + (n_{\text{nbr1}}/(\sigma n_{\text{titin}}))^{-m_{\text{nbr1}}}} = \text{const.} \quad . \tag{22}$$

In the simplified case where nbr1 availability does not markedly change over time (because the number of signalling complexes remains small), the Hill equation will only be important in setting the steady-state concentrations and force in the muscle.

PART B: SIGNAL LEADING TO PROTEIN SYNTHESIS

Part B.1: Estimate of the relative molecular abundances

Sarcomere lattice dimensions

The distance between neighbouring filaments can be estimated from X-ray diffraction measurements[33–36] as $d_{mm} \approx$ 46nm for a resting sarcomere length of $\approx 2.3\mu$m, under no tension and for a range of different organisms. As the titin passive tension increases (*i.e.* during the extension of the sarcomere during exercise), the inter-filament distance decreases approximately linearly with tension, as measured by[81], while the sarcomere length increases roughly quadratically with tension[82].

Molar density of titin

There are 12 titin molecules between neighbouring Z-discs per thick filament[83–85]. This means that the molar density of titin within the contractile myofibrils is essentially constant throughout a range of different muscle types: $[\text{Ttn}] \approx 4.7\mu$M .

Molar density of ATP and creatine phosphate

The concentrations of ATP, ADP and AMP as well as creatine (Cr) and phosphocreatine (PCr) have been accurately measured during exercise in human muscle by[86]. By summing the individual contributions from ATP+ADP+AMP and PCr+Cr, we can determine their maximum concentration to be (adjusting to wet weight from dry weight by a factor of approximately 4.0 ± 0.1[87]: $[\text{ATP}]_{\max} \approx 7.2 \pm 0.3$mM and $[\text{Cr}]_{\max} \approx 29 \pm 1$mM.

Molar density of G-actin

The thin filament length varies widely between muscle types and can extend further than the free length of $\approx 0.9\mu$m if is stabilised by nebulin[88]. If we take an intermediate value of 1.2μm (range $0.9 - 1.4\mu$m) for skeletal muscle (where F-actin is definitely stabilised by nebulin), then we can estimate the number of G-actin monomers per sarcomere.

The thin actin filament contains two helically-arranged F-actin chains. The axial distance between two G-actin monomers on one strand is some 5.5nm[89]. Each F-actin polymer therefore contains approximately 220 (range $160-260$) G-actin monomers. The ratio of F-actin to titin molecules is 2 to 3, so the molar density of G-actin is $\approx 700\mu$M (range $\approx 550 - 800\mu$M , liberally rounding).

Molar density of the signaling molecules

We used a combination of relative mRNA data and degradation rates to estimate the relative concentrations of titin to nbr1 to lie between 100 and 1000. This suggests that the molar density of nbr1 is of the order of $\approx 0.005 - 0.05\mu$M .

We will assume for simplicity that the ratio of titin to nbr1 remains constant. If their concentration is determined by the concentration of ribosomes as we suggest here, then it seems plausible that both proteins could be synthesised in similar ratios regardless of the size of the cell.

Molar density of ribosomes

RNA makes up only ca. 4% of the dry weight of a typical mammalian cell[90]. Of this, some 80% is rRNA, and some 85% of rRNA is incorporated into ribosomes. As such, ca. 1% of the wet weight of the typical cell is found in ribosomal rRNA, and perhaps near 2% in ribosomes, which also include ribosomal proteins[91].

In myocytes, a large portion of proteins are structural, and have longer half-lives than those of many other cells[92], so it seems likely that the ribosomal weight is lower, perhaps only 1% of the wet weight of the cell.

Each ribosome has a molecular weight of $3.2 \cdot 10^6 \mathrm{g\,mol}^{-1}$, so the number of ribosomes works out at $\approx 3\mu\mathrm{M}$. We will check that we have an approximate ratio of titin to ribosomes of the order of $1 - 2$ below.

The number of DNA molecules does not increase without myonuclear accretion, which is not included in our model. Rather than keeping track of the relative decrease of DNA concentration as the cell grows, we consider the total number of molecules in the cell. As the cell grows, the number of signalling molecules produced increases in proportion with the size of the cell. These all appear to end up in the cell's nucleus ([68]), so it seems plausible that an increase in the total number of activated SRF molecules in the cell leads to a proportional increase in the activation of RNA synthesis, regardless of the size of the cell. If RNA synthesis then increases linearly with the activation of the DNA promoter (discussed in relation with SRF below), then we can simply write a kinetic equation for the concentration of rRNA (*i.e.* roughly similar to the ribosome concentration) in terms of a synthesis term proportional to the concentration of SRF and a degradation term.

Part B.2: Ribosome biogenesis regulates synthesis of new proteins

A growing muscle cell must increase its biosynthetic activity in order to maintain homeostasis. Because the cell spends most of its biosynthetic budget on making ribosomes[93,94], and because the cell's growth rate is known to be strongly influenced by its ribosome content[95,96], it seems likely that ribosome number is already a limiting factor for protein synthesis in the muscle prior to resistance training – anything else would be very energy inefficient. Indeed, ribosome biogenesis has recently been suggested to be necessary for skeletal muscle hypertrophy to occur[97–102]. As the cell's biosynthetic demands increase during resistance training, ribosome number must therefore increase before an increase in mRNA coding for various sarcomeric proteins can actually translate to an increase in protein synthesis.

Protein synthesis is raised immediately after exercise, indicating an increase in RNA Polymerase II activity. This surplus peaks at 24 hours, before essentially returning to normal levels by 36 hours[103]. The increase in muscle protein synthesis results from a combination of an uptick in mRNA synthesis and an activation of protein translation (*e.g.* by mTOR). At the same time, protein degradation increases after exercise, and there is only a small net positive synthesis of proteins in hyperaminoacidemic conditions[104]. Because of this, it is reasonable to add protein synthesis and degradation together, and to ignore short-term fluctuations in the muscle's protein balance.

As the resistance training programme progresses, there can only be a sustained net increase in protein number if the rate-limiting number of ribosomes increases (excluding the short term adaptations immediately following a single exercise session: intermittent bouts of increased mRNA and translational activation). To a first approximation, protein synthesis rates will be proportional to ribosome number, whose synthesis by RNA Polymerase I will in turn be proportional to the mechanosensitive signal strength.

Part B.3: Signal, ribosome and sarcomere protein synthesis & degradation rates

In the light of the above reasoning, we need to update our equations to include SRF (let us assume that this is still the signal s, more on this later), ribosomes and titin (used as a proxy for all sarcomere structural proteins, which must be synthesised in concert). Together, these equations help account for the lag between starting resistance training and the onset of muscle hypertrophy several weeks later. But first, we need to estimate their synthesis and degradation rates and check to see if our model predicts a correct ballpark value for the relative concentration of signal, ribosomes and titin in the steady-state.

- Recent single cell experiments by[105] showed that starved 3T3 cells contained of the order of 20 SRF molecules which bind to DNA for long periods of time. The volume of NIH 3T3 cells is of the order of 2.4 pL[106], compared

with a typical myonuclear volume of muscle cells at 16 pL. If SRF is truly required to activate ribosome biogenesis, then a simple scaling (assuming similar molecular constituent degradation rates) would suggest that the typical muscle fibre sarcomere cross-section (2.3 μm in length with an area of $4000 \mu m^2$: volume $\approx$ 9 pL) would contain of the order of 100 SRF molecules which bind for long periods of time to DNA: *i.e.* activated phospho-SRF molecules. This result is consistent with our prediction of the steady-state SRF concentration using the kinetic rate constants in our model (see Part D below).

- The peak rRNA synthesis rate must be nearly reached in exponentially growing budding yeast. Experiments by French et al.[107] showed that the distance between neighbouring RNA Pol I in budding yeast was typically 132 nucleotides. Perez-Ortin et al.[108] measured a maximum transcription speed for RNA Pol II in yeast (assumed similar to that of RNA Pol I) of 42nt s^{-1}. Together, this tells us that rDNA translation initiation occurs at a highest rate of $0.32 s^{-1}$.

  A typical synthesis rate should be a little lower, so we choose $k_{sr} = 0.1 s^{-1}$ as a typical ribosome synthesis rate per activated rDNA repeat. Multiplying this value by the number of active SRF molecules per myonuclear domain ($\approx$ 150, scaled to a volume of 16 pL) predicts that some 15 ribosomes per second are synthesised per myonuclear domain. The next paragraph is an aside to check if this value is in the correct ballpark.

  Comparison between muscle and liver ribosome transcription rates: Homo sapiens has 350 rDNA repeats[109] (700 on both pairs of chromosomes), which in turn means that there is a maximum synthesis rate of $\approx$ 200 ribosomes s^{-1} per nucleus. Protein degradation rates are quite different in different tissues:[92] show for instance that skeletal muscle degrades almost 4 times slower (28.6 day protein half-life) than liver tissue (7.35 day protein half-life). Correspondingly, we expect ribosome biogenesis to differ by a similar factor in both tissues. Ribosome turnover rates have long been known to be high in the rat liver ([110] measured ribosome synthesis rates of over 1000 ribosomes $\min^{-1}$ nucleus^{-1}). The volume of rat hepatocytes has been reported at $\approx 3.68 \pm 1.37$ pL[111], a factor of 4-5 less than the myonuclear volume. Estimating the differential rate of ribosome synthesis to be roughly proportional to the differential protein half-life yields a 4 times lower maximum synthesis of ribosomes *per unit volume* in skeletal muscle. Scaling to a value per nucleus gives suggests an estimate of ca. 20 ribosomes synthesised per second and per nucleus for typical muscle cells. This is more than 10 times less than the maximum possible synthesis rate of ribosomes and indicates that significant upregulation of this number can occur in the presence of transcription factors such as SRF. In addition, this estimate is consistent with the ballpark value for ribosome synthesis obtained from our model's parameters.

- Ribosome degradation rates have been well-estimated in many tissues including liver[112,113] and brain[110]. Unfortunately, measurements in skeletal muscle are less clear. The clearest measurement that we could find was of pre-diabetic rats by Ashford and Pain[114,115]. The rats that they considered were still growing at the start of the experiment, meaning that their rRNA degradation rates were too low and their rRNA synthesis rates too high. But their data suggests that skeletal muscle rRNA has a fractional turnover rate of $\approx 5\%$ per day (with a range of $2-7\%$): the control sample (still growing) shows a synthesis rate of 7% per day, while the very low degradation rate is almost unnoticeable at $< 2\%$. Diabetic rats on the other hand show very high rRNA degradation rates until insulin is provided and their degradation rates revert to $2-4\%$ per day. We use these combined observations to estimate the actual turnover rate at $\approx 5\%$ per day, or a half-life of ≈ 14 (range $10-30$) days. This is somewhat slower than the rRNA turnover rate in liver (≈ 0.1day^{-1}[113]), as we would expect in a tissue with lower turnover. Clearly, this rate would benefit from more detailed observations.

- Titin synthesis rates k_{st} require another estimate. Neglecting the correlation between protein size and degradation rates in all proteins apart from the sarcomere proteins (*e.g.*[116] highlight an inverse correlation between protein size and susceptibility to lysosomal degradation), we can use information about the fractional masses of sarcomeric structural proteins and their lifetimes to consider the fraction of ribosomes which must be used to synthesise titin.

  Titin accounts for ca. 10% of the myofibril mass[117,118], and has a half-life of ca. 3 days[75]. Myosin accounts for ca. 43% of the myofibril mass and has a very long half-life of up to 30 days[119]. Finally, actin makes up ca. 22% of the myofibril mass and also has quite a long half-life in muscle[120,121] at 10.3 days. In other words, titin turnover is fast relative to the other muscle constituents, and consequently we would expect titin amino acids translated in similar proportions to actin and myosin amino acids.

  Assuming that the other muscle proteins are degraded faster at an average rate of 43 hours^{-1}[122], that they make up 25% of the sarcomere and all of the other muscle's organelles, such that sarcomere structural proteins only make up ca. 60% of the muscle mass, then we see that some $10-15\%$ of translation events are likely titin-related.

Ribosomes translate new proteins with a rate of 6-9 amino acids per second[123]. Titin has some 34000 amino acids, so it takes one ribosome $4000-6000$ seconds to translate one titin molecule. Consequently, we can estimate the rate of titin synthesis to be $k_{st} \approx 10^{-5}s^{-1}$ (accounting ca. 90% of ribosomes not synthesising titin and for some unbound ribosomes).

- **Consistency check:** The estimates in this section give $n_{\text{rRNA}} = n_{\text{SRF}} \cdot k_{sr}/k_{dr} \approx 10^7$ ribosomes per sarcomere cross-section (length 2.3μm, area 4000μm^2) and $n_{\text{titin}} = n_{\text{rRNA}} \cdot k_{st}/k_{dt} \approx 2.5 * 10^7$ titins per sarcomere cross-section. That value is very close to the actual number of titins in a cross-section of length 2.3μm and area 4000μm^2.

NB: None of these rates significantly impact the overall qualitative mechanosensitive behaviour of TK, so rough estimates are acceptable. The main impact which they will bring about is to change some of the time scales on which the system responds; in particular, the ribosome degradation rate is important in determining both the lag time until muscle hypertrophy is observed and the atrophy rate of the muscle - because it is substantially slower than the muscle protein degradation rate.

Part B.4: Ribosomes are degraded en-route to the sarcomere; muscle size feedback

New experiments tracking titin synthesis and integration into the sarcomere show that most titin is synthesised by ribosomes which localise to the sarcomere M-band or Z-disk[124]. This means that titin mRNAs must be transported from the nucleus to the sarcomere prior to protein synthesis. Titin mRNA is very massive (81000 nt or 26.7 MDa), so it will be very affected by diffusive constraints.[125] However, Rudolph et al.[124] suggest that titin mRNA does not diffuse freely in the cell and rather localises along sarcomeric tracks. In other words, diffusive constraints might not be quite as severe for titin mRNA as extrapolated from the diffusion of other large proteins.

Ribosomal subunits are manufactured in the nucleolus and have to diffuse to the sarcomeric synthesis sites, namely the M-band and Z-disk. Ribosome subunits can be approximated to diffuse much like globular proteins; under this approximation, the diffusion of the large subunit ($\approx$ 2 MDa in eukaryotes) is the limiting step. The diffusion of globular proteins is known to be strongly inhibited by the spacing of the myofibrillar lattice, which acts as a sieve. In particular, extrapolating the data obtained by Papadopoulos et al.[126] concerning the diffusion coefficients of large globular proteins in the sarcoplasm suggests that the diffusion coefficient of the large ribosomal subunit should be of the order of $10^{-2} - 10^{-1}\mu\text{m}^2\text{s}^{-1}$, or perhaps even smaller. With such a diffusion constant, the ribosomal subunit would take $t \approx x^2/(6D) \approx 10^3 - 10^4\,\text{s}$ to diffuse across a typical myonuclear domain (volume $\approx 16000\mu m^3$, typically $20-25\mu$m from the edge of the myotube where the nucleus is found to the opposite edge of the myonuclear domain at the center of the tube). The half-life of ribosomes is $100-1000$ times higher, so given this simple extrapolation of the ribosomal diffusion constant, $0.1-1\%$ of the total ribosomes are degraded before they reach their target positions within the sarcomere. But given the sieve-like nature of the myofibrillar lattice, we suspect that the actual diffusion constant of ribosomes is much lower. Pending better estimates of sarcoplasmic ribosome diffusion, we will treat it as a variable.

This term, which represents the en-route degradation of ribosomes, must depend on the myonuclear domain size: if the muscle cell grows, ribosomes must move further and are more likely to be degraded. The diffusion time depends on the square of the distance, and so does the number of actin filaments. To a first approximation, this degradation term is proportional to the myofibre CSA or to the total number of titin filaments. The effective ribosome synthesis rate is reduced from that which would be expected if all of the ribosomes could be instantaneously transported to the sarcomere by a factor $(1-\alpha n_{\text{titin}})$, where the proportionality constant α depends on the sarcoplasmic diffusion coefficient of the ribosomal subunit.

We see in the main text that the diffusive constraints on ribosome transport in muscle contained in this term provide the necessary feedback to stop runaway cell hypertrophy in our model.

PART C: PHOSPHATE KINETICS LIMIT TK SIGNALLING

Part C.1: Exercise intensity and duration affects the signalling ability of TK

In order to consider exercise at a higher intensity, and which lasts for more than a few seconds, it is necessary to include in our equations some knowledge of how the supply of free ATP changes during exercise. The duration of most exercise sets aimed at inducing muscle hypertrophy is at least a few minutes[127]. During this time, the body's fast energy stores are rapidly depleted[128]: free cellular ATP degradation increases and leads to a near-equal synthesis of new ATP from creatine phosphate (which accounts for slightly more than four times that amount[129]) to maintain stable ATP levels. Glycogen stores then begin to be used when CP stores become depleted: readily available glycogen is converted to glucose via glycogenolysis and to ATP via oxidative phosphorylation as the body prepares for longer-distance exercise. Together, these mechanisms allow for a graded response to different effort durations and intensities. At very long timescales (beyond the scope of resistance exercise or everyday activity), glycogen stores become depleted, and fatty acid oxydation become provides significant and serves as the main energy store for ultra distance efforts[130].

At medium to long timeframes and lower intensities, energy levels within the muscle are limited by the rate of oxygen uptake, which itself determines the rate of mitochondrial oxidative phosphorylation[131,132].

Both phosphocreatine kinetics and oxidative phosphorylation kinetics may impact the availability of ATP for the phosphorylation of signalling sites. In the TK-mediated mechanosensing paradigm, the phosphorylation of TK would be expected to be lower when exercise is too hard, through a combination of decreased ATP availability through the phosphocreatine system or via lack of ATP production from mitochondria. This effect is apparent in Fig. 9 in the main text.

Part C.2: Kinetic equations for ATP/CP creation and consumption

The synthesis, degradation and transfer kinetics of the main metabolic species, namely ATP, CP and glycogen, are well known.

Glycogen stores are fully depleted after several hours of exercise[133], and the interplay between glycogen and fatty acid metabolism is complex and likely depends strongly on the exercise history of the individual in question. Rather than going into the complexities of this question, we will simply consider types of exercise where the distinction is unnecessary, either as the exercise occurs faster than the maximal uptake rate of oxygen (VO2max), or where the exercise is at a sufficiently low intensity (i.e. every day activity) that energy store type is not a rate limiting step in ATP synthesis.

In order to determine the concentration of ATP available for phosphorylation, we need to keep track of the processes which consume, synthesise or transform energy. Neglecting glycogen store size, we therefore only need to consider ATP and CP concentrations.

The following rates must be present in the kinetic equations:

- Consumption of ATP by the muscle: this is proportional to the number of myosin molecules, the binding fraction of each myosin molecule on the thin filament (the myosin duty ratio, which is measured to be close to 0.05[48]), the force of ≈ 5.7pN produced by myosin stepping from rest due to the consumption of one ATP molecule[42,134], and the time which it takes myosin II to go though a single ATP cycle at $\approx 135ms$ at a roughly physiological 2 mM concentration of ATP[46].

  The myosin head performs work early in the binding interval[135], meaning that it applies the force of ≈ 5.7pN for much of the binding time, including after the end of the working stroke. The binding time is of the order of 7ms as determined from the product of the duty ratio with the ATP cycle. To apply a force of ≈ 5.7pN on average, one ATP molecule must therefore be consumed every 7ms. This gives a constant of $k_{\text{myo}} \approx 2 \cdot 25\ \text{s}^{-1}\text{pN}^{-1}$ ATP molecules consumed per second and per pN of constant load per sarcomere (where the factor of 2 accounts for the symmetry of the sarcomere on either side of the M-line). ATP consumption per sarcomere therefore changes with a rate $-k_{\text{myo}} f_{\text{fibre}} = -r k_{\text{myo}} f_{\text{titin}} n_{\text{titin}}$ where we have included a factor $r = (f_{\text{filament}}/6 f_{\text{titin}})$ to account for the fact that the force is spread out between the 6 parallel titins and myosin. Titin kinase is most sensitive if r is small, and we $r = 3$ as a plausible value (based on the approximate dependence of TK force on myosin force found in Part A.4; see in particular Fig. 3). In our model, we consider the number of proteins within a ca. 2μm thick cross-section of a muscle fibre, and this term accounts for the ATP consumption within such a slice.

- ATP can be synthesised from CP with a rate $k_{\text{CP}\to\text{ATP}} \frac{([\text{ATP}]_{\max} - [\text{ATP}]_{\text{free}})}{[\text{ATP}]_{\max}}[\text{CP}]$. The rate of creatine phosphate conversion to ATP must depend on the need for more ATP. During an all-out sprint, creatine phosphate stores

are diminished by a factor of ≈ 4 after 30 seconds of all-out exercise[136]. In other words, the fastest rate of conversion of CP to ATP must be of the order of 1-2 mM s^{-1}. ATP is buffered by CP and does not fall below 70% of resting values[137], meaning that the rate $k_{\mathrm{CP}\to\mathrm{ATP}} \approx 0.1 - 0.2\mathrm{s}^{-1}$.

- CP can be synthesised from ATP with a rate $k_{\mathrm{ATP}\to\mathrm{CP}} \frac{([\mathrm{CP}]_{\max}-[\mathrm{CP}])}{[\mathrm{CP}]_{\max}} [\mathrm{ATP}]_{\mathrm{free}}$. The recovery of phosphocreatine after exercise has been shown to be well-described by a biphasic exponential of the following form[138]:

$$[CP(t)] = CP_{\max} - \left(c_1 e^{-k_{\mathrm{faster}} t} + c_2 e^{-k_{\mathrm{slower}} t}\right) \quad . \tag{23}$$

Concerning the initial recovery of CP up to $70-80\%$ of its maximum value (expressed by the fast exponential in Eqn. 23), we can solve for $k_{\mathrm{ATP}\to\mathrm{CP}}$ by assuming that the rate of change of CP is much faster than the rate of change of ATP (which contributes to the slow exponential in Eqn. 23), so that the phosphocreatine equation is in a local steady-state:

$$\frac{d[CP(t)]}{dt} \approx 0 \Rightarrow k_{\mathrm{ATP}\to\mathrm{CP}} \approx k_{\mathrm{CP}\to\mathrm{ATP}} \frac{\frac{([\mathrm{ATP}]_{\max}-[\mathrm{ATP}]_{\mathrm{free}})}{[\mathrm{ATP}]_{\max}}}{\frac{([\mathrm{CP}]_{\max}-[\mathrm{CP}])}{[\mathrm{CP}]_{\max}}} \frac{[\mathrm{CP}]}{[\mathrm{ATP}]_{\mathrm{free}}} \tag{24}$$

This can then be evaluated at the end of a sprint; for the sake of simplicity, we use the values measured by[139], and find: $k_{\mathrm{ATP}\to\mathrm{CP}} \approx 0.15 - 0.3\ \mathrm{s}^{-1}$.

- Rate $k_r n_p$ of phosphate return from TK phosphorylation (minimal): mostly due to phosphorylated TK converting to open TK. Many other signalling pathways would also yield such an effect.

- The rate of ATP synthesis from oxidative phosphorylation depends primarily on the maximum rate of oxidative phosphorylation k_{O_2} which is proportional to VO2max in the following manner. The breakdown of each glucose molecule requires 6 O2 molecules and generates a theoretical maximum of 38 ATP molecules (but an actual maximum closer 29-30 ATP[140]). Every litre of oxygen contains $\approx 2.7 \cdot 10^{22}$ molecules at standard pressure and temperature. This can therefore generate up to $29.5/6 \cdot 2.7 \cdot 10^{22} \approx 1.3 \cdot 10^{23}$ ATP molecules per second. The record rate of oxygen consumption in muscle is approximately 100 ml min^{-1} kg^{-1}[141], which should be scaled by a factor of $\approx 2 - 2.5$ to account for muscle mass only making up $\approx 40 - 50\%$ of body mass at most, in an age-dependent manner[142]. The muscle would then produce of the order of $5 \cdot 10^{20}$ ATP molecules s^{-1} kg^{-1}. We obtain the corresponding estimate per titin molecule scaled by the $\approx 4.7 \cdot 10^{-6} \cdot 6.02 * 10^{23} \approx 2.8 \cdot 10^{18}$ molecules of titin in each kg of muscle (using the result from Part B.1 above). This gives a world-record rate of $k_{\mathrm{O}_2} \approx 200$ ATP molecules per titin molecule generated per second, and a normal rate for amateur athletes of perhaps half that[143].

As the ATP deficit increases, we suggest that the rate of O_2 uptake increases linearly to begin with before plateauing when the VO2max limit is reached. A simple way to extrapolate this behaviour into a non-piecewise function is with the following term:

$$k_{\mathrm{O}_2} \frac{\frac{([\mathrm{ATP}]_{\max}-[\mathrm{ATP}]_{\mathrm{free}})}{[\mathrm{ATP}]_{\max}}}{\left(\left(\frac{([\mathrm{ATP}]_{\max}-[\mathrm{ATP}]_{\mathrm{free}})}{[\mathrm{ATP}]_{\max}}\right)^e + k_{\mathrm{shape}}\right)^{1/e}} n_{\mathrm{titin}} \tag{25}$$

where k_{shape} and e are empirical shape-fitting constants. While it looks complicated, this equation is just a linear term depending on the ATP deficit: $\propto \frac{([\mathrm{ATP}]_{\max}-[\mathrm{ATP}]_{\mathrm{free}})}{[\mathrm{ATP}]_{\max}}$ followed by a constant term, with a more or less smooth transition zone between the two, whose shape depends on the two constants k_{shape} and e.

- All of these rates were scaled relative to the steady-state (maximum) values of ATP or CP listed in Part B.1 above.

Combining the above rates yields two additional kinetic equations for free ATP and creatine phosphate (CP):

$$\begin{aligned}\frac{d[\mathrm{ATP}]_{\mathrm{free}}}{dt} &= (-r k_{\mathrm{myo}} f_{\mathrm{titin}}[\mathrm{Ttn}] + k_{\mathrm{CP}\to\mathrm{ATP}} \frac{([\mathrm{ATP}]_{\mathrm{max}}-[\mathrm{ATP}]_{\mathrm{free}})}{[\mathrm{ATP}]_{\mathrm{max}}}[\mathrm{CP}] \\ &+ k_{\mathrm{O}_2} \frac{\frac{([\mathrm{ATP}]_{\mathrm{max}}-[\mathrm{ATP}]_{\mathrm{free}})}{[\mathrm{ATP}]_{\mathrm{max}}}}{\left(\left(\frac{([\mathrm{ATP}]_{\mathrm{max}}-[\mathrm{ATP}]_{\mathrm{free}})}{[\mathrm{ATP}]_{\mathrm{max}}}\right)^{e} + k_{\mathrm{shape}}\right)^{1/e}}[\mathrm{Ttn}] \\ &- k_{\mathrm{ATP}\to\mathrm{CP}} \frac{([\mathrm{CP}]_{\mathrm{max}}-[\mathrm{CP}])}{[\mathrm{CP}]_{\mathrm{max}}}[\mathrm{ATP}]_{\mathrm{free}} - k_p n_o [\mathrm{ATP}]_{\mathrm{free}} + k_r n_p\end{aligned} \tag{26}$$

$$\begin{aligned}\frac{d[\mathrm{CP}]}{dt} &= -k_{\mathrm{CP}\to\mathrm{ATP}} \frac{([\mathrm{ATP}]_{\mathrm{max}}-[\mathrm{ATP}]_{\mathrm{free}})}{[\mathrm{ATP}]_{\mathrm{max}}}[\mathrm{CP}] \\ &+ k_{\mathrm{ATP}\to\mathrm{CP}} \frac{([\mathrm{CP}]_{\mathrm{max}}-[\mathrm{CP}])}{[\mathrm{CP}]_{\mathrm{max}}}[\mathrm{ATP}]_{\mathrm{free}} \quad .\end{aligned} \tag{27}$$

These equations naturally assume that the number of muscle sarcomere proteins scales with the total number of titin molecules, *i.e.* that the composition of the sarcomere remains the same throughout trophic changes.

TABLE I: Values of rate constants for phosphate transfer, obtained in experiments or simulations.

Constant	Value (s^{-1})	Source
k_{myo}	$2.4 \cdot 10^{-4}\mathrm{pN}^{-1}$	[42,48,134,135 a]
$k_{\mathrm{CP}\to\mathrm{ATP}}$	0.1-0.2	[136,137]
$k_{\mathrm{ATP}\to\mathrm{CP}}$	0.15-0.3	[136,137,139]
k_{O_2}	100	[141,143]
k_{shape}	10^{-40}	[b]
e	50	[c]

None of the sources directly report these values, so one should followthe discussion in this sectionfor details about how they were obtained.

[a] Value per pN of titin force.

[b] For a very sharp transition from linear to constant regime, no data. Conversely, e needs to be quite large.

[c] For a very sharp transition from linear to constant regime, no data. Conversely, k_{shape} needs to be very small.

PART D: STEADY-STATE SOLUTION FOR THE FULL MODEL

All of the equations in the main text, together with the phosphate equations from the previous section, yield a steady-state solution. Unfortunately, one of the phosphate equations does not possess an analytical solution. Its steady-state solution can be found most easily by asymptotically tending towards a steady-state by iteratively changing the values for free ATP and CP (not the maximum values, which are fixed model parameters).

Let us solve for the steady-state concentrations of the other molecular species:

$$n_c = \frac{k_- k_{dn_s}\left(k_r + \tilde{k_s}\right)}{\Phi_1} \ , \quad n_o = \frac{k_+ k_{dn_s}\left(k_r + \tilde{k_s}\right)}{\Phi_1} \ , \quad n_p = \frac{k_+ k_{dn_s} k_p[\mathrm{ATP}]}{\Phi_1} \ , \quad n_s = \frac{k_+ \tilde{k_s} k_p[\mathrm{ATP}]}{\Phi_1} \tag{28}$$

where

$$\Phi_1 = k_+\left(k_{dn_s}\left(k_p[\mathrm{ATP}] + k_r + \tilde{k_s}\right) + \tilde{k_s}k_p[\mathrm{ATP}]\right) + k_- k_{dn_s}\left(k_r + \tilde{k_s}\right) \tag{29}$$

and

$$\tilde{k_s} = \frac{k_s}{1 + (n_{\mathrm{nbr1}}/(\sigma n_{\mathrm{titin}}))^{-m_{\mathrm{nbr1}}}} \quad . \tag{30}$$

The equations for SRF, ribosomes and titin can easily be combined and solved for a steady state for SRF, ribosomes and mechanosensing complex numbers n_s:

$$n_{\mathrm{SRF}} = \frac{k_{dt}k_{\mathrm{dr}}}{k_{st}k_{\mathrm{sr}}} n_{\mathrm{titin}} \ , \quad n_{\mathrm{rRNA}} = \frac{k_{dt}}{k_{st}} n_{\mathrm{titin}} \ , \quad n_s = \frac{k_{dt}k_{\mathrm{dr}}k_{\mathrm{ds}}}{k_{st}k_{dn_s}k_{\mathrm{sr}}} n_{\mathrm{titin}} \ . \tag{31}$$

The steady-state nbr1 concentration can be obtained simply by combining the equations for nbr1 (Eqn. 22), and the above equations for the mechanosensing complex and ribosomes in terms of the titin concentration (Eqns. 31):

$$n_{\text{nbr1}} = \frac{k_{dt}}{k_{d,\text{nbr1}}}\Big(\gamma_{\text{nbr1/Ttn}} - 2\frac{k_{d,\text{ribo}}k_{ds}}{k_{\text{sribo}}k_{st}}\Big)n_{\text{titin}} \tag{32}$$

where $\gamma_{\text{nbr1/Ttn}} = k_{s,\text{nbr1}}/k_{st} \approx 0.002$ (see Part A.7) accounts for the relative synthesis rates of nbr1 to titin. Finally, Eqns. 26 and 27 combine to give a steady-state CP and ATP concentration. Combining Eqns. 28 and 31, we can solve for the steady-state force, inserting the relations found above for the opening and closing rates k_- and k_+:

$$f_{\text{st}} = \frac{\Delta G_0}{u_{\max}} + \frac{k_BT}{u_{\max}}\ln\Big(-\frac{(k_r + k_s')}{k_p[\text{ATP}]\big(\frac{k_s'}{k_{dn_s}} - \zeta + 1 + \frac{k_r + k_s'}{k_p[\text{ATP}]}\big)}\Big) \tag{33}$$

where 'st' stands for steady-state, the shorthand ζ is the ratio of synthesis to degradation coefficients:

$$\zeta = \frac{k_{st}\tilde{k_s}k_{sr}}{k_{dt}k_{dr}k_{ds}}\ , \tag{34}$$

and the shorthand $\tilde{k_s}$ contains information about the aggregation dynamics of the mechanosensor:

$$\tilde{k_s} = \frac{k_s}{1 + (n_{\text{nbr1}}/(\sigma n_{\text{titin}}))^{-m_{\text{nbr1}}}} = \frac{k_s}{1 + \Big(\sigma\frac{k_{dt}}{k_{d,\text{nbr1}}}\Big(\gamma_{\text{nbr1/Ttn}} - 2\frac{k_{d,\text{ribo}}k_{ds}}{k_{\text{sribo}}k_{st}}\Big)\Big)^{-m_{\text{nbr1}}}}\quad . \tag{35}$$

PART E: MISCELLANEOUS

Part E.1: Non-linear dependence of force on muscle size

$$f = F/n_{\text{titin}}\ ,\quad F = F_0\Big(\frac{n_{\text{titin}}}{n_{\text{titin}}(F = F_0)}\Big)^{1-\mu} \quad \Rightarrow f = f_0\Big(\frac{n_{\text{titin}}}{n_{\text{titin}}(F = F_0)}\Big)^{-\mu}\ . \tag{36}$$

We also consider the possibility that the force produced by the muscle does not scale linearly with muscle size ($\mu = 0$). It is unclear exactly how much active muscle force scales with muscle size. Krivickas et al.[37] find that force increases slower at larger muscle CSA, whereas Akagi et al.[144] do not see a substantial non-linearity between force and myofibre volume. This suggests that the apparent non-linearity between muscle force and muscle fibre size has more to do with a change in muscle architecture and cross-sectional thickness as pennation angles increase during hypertrophy. It is tempting to look for an explanation for trophic feedback in terms of tendon elongation or shortening in response to hypertrophy or atrophy respectively. However, increases in tendon stiffness and long-term tendon hypertrophy are well-documented in conjunction with myocyte hypertrophy, and so is a decrease of tendon stiffness during muscle atrophy[38,145]. So, it is also not clear if there is any significant muscle passive force feedback. We consider the implications of both types of feedback in the main text.

Part E.2: Reversible mechanosensor transition rates

The transition rates between the aforementioned titin conformations involve the opening and closing of protein domains. Such a scenario was analysed by[32] for FAK, who found that both kinds of rates had a complex dependence on the tension within the molecule,

$$k_+(f) = \Big(\frac{\Delta G_{0,a}}{u_{0,a}^2\gamma_{\text{TKD}}}\Big)\frac{g_a\bar{f_a}^2(1 - 2\bar{f_a}/3)^2\zeta}{4(1 - 2\bar{f_a}/3)^2\Psi_{1,a}[f] + \bar{f_a}^2\zeta\Psi_{2,a}[f]} \tag{37}$$

where the functions $\Psi_{1,a}[f]$ and $\Psi_{2,a}[f]$ are shorthand for:

$$\Psi_{1,a}[f] \quad = \exp\big[-g_a\bar{f_a}^2/2\bar{\kappa_a}(f)\big] + g_a\bar{f_a}^2/2\bar{\kappa_a}(f) - 1 \tag{38}$$

$$\Psi_{2,a}[f] = \exp\big[g_a(1 - 2\bar{f_a}/3)^{3/2}\big] - g_a(1 - 2\bar{f_a}/3)^{3/2} - 1 \tag{39}$$

and where the energy barrier $g_a = \Delta G_{0,a}/k_B T$, the force $\bar{f}_a = f \times u_{0,a}/\Delta G_{0,a}$, stiffness of the muscle fibre $\bar{\kappa_a}(f) = \kappa(f) \times u_{0,a}^2/\Delta G_{0,a}$ and the ratio of damping constants $\zeta = \gamma_{\rm TKD}/\gamma_{\rm fibre}$ have been made dimensionless.

The rate of auto-inhibition on the other hand does not depend on the substrate stiffness and can be found as with FAK to be:

$$k_-(f) = \Big(\frac{\Delta G_{0,a}}{u_{0,a}^2 \gamma_{\rm TKD}}\Big) \frac{g_a \bar{f}^2}{\big(e^{g_a \bar{f} \lambda_a} - 1\big) - g_a \bar{f} \lambda_a} \tag{40}$$

where the $\lambda_a = (u_{\max,a} - u_{0,a})/u_{0,a}$ is the ratio of the lengths of the open and closed conformations. Very similar relations can be obtained for the other rates involving TK conformational change, *i.e.* those for $k_c, k_e, k_{e'}$.

Most of the physiological parameters can be obtained directly from AFM experiments by Puchner et al.[58]. The transition energies G_0 can be obtained from the area below the curve in the force-displacement relationship plotted in their Figure 3. If our attribution of the transitions between the different TK conformations is correct, then the energy barrier between the closed and the open state is of the order of $G_{0,a} \approx 50 k_B T$. The energy difference between the transition from the open to the over-extended conformations in the absence of ATP, $G_{0,b}$, is slightly smaller to that seen in the presence of ATP ($G_{0,c}$), with perhaps $G_{0,c} - G_{0,b} \approx 30 k_B T$ and $G_{0,b} \approx 200 k_B T$. From their graph, setting the zero point of the problem as the partially unfolded structure (but still closed) at $\approx$ 19nm, the first peak is at $u_{0,a} \approx$ 1.5nm and the next peak is at $u_{\max,a} \approx$ 7nm. For the next peak, again setting the zero point at the through between the two peaks, $u_{0,b} \approx u_{0,c} \approx$ 4.5nm again and $u_{\max,b} \approx u_{\max,c} \approx$ 9nm .

The stiffness of the muscle scales with the tension force within the muscle as shown for instance by[146] due to the rearranging of components during loading, including the Z-disk cross-bridges. The relationship between muscle force and its Young modulus was examined by[147], who found a linear relationship between torque and stiffness up to the maximum isometric torque. The measured Young's modulus at low force is quite small, so we can assume that the Young's modulus increases linearly with a negligible constant as

$$E(f) \approx E_{\max} \frac{f}{f_{\max}} \tag{41}$$

where the measured $E_{\max} \approx$ 200 kPa. The stiffness of the substrate of the titin fibre can be estimated by considering the Z-line as a plane and taking the stiffness of the Z-line to be the same as that of the muscle. The stiffness of the titin substrate is then given as $\kappa(f) = (4/3)\pi E(f)\eta$, where $\eta \approx$ 50nm is an elastic cutoff corresponding to the mesh size between neighbouring force-bearing filaments. This gives an estimate of a maximal titin substrate stiffness of $\kappa_{\max} \approx 0.04\,{\rm N\ m}^{-1}$.

Assuming that the internal diffusion constant of TK is similar to that of FAK gives an estimate of the damping constant of TK as: $\gamma_{TK} \approx 10^{-9}{\rm kg\,s}^{-1}$. Bell et al.[32] find that the substrate damping constant depends on its fluctuation relaxation time $\tau_{\rm sub} \approx 0.01$s which is quite universal across wide ranges of substrate stiffness. This gives the dissipation constant for the substrate as $\gamma_{\rm fibre} \approx 4 \cdot 10^{-4}{\rm kg\,s}^{-1}$, and so the ratio $\zeta = \gamma_{\rm TKD}/\gamma_{\rm fibre} \approx 10^{-5}$.

Part E.3: Phosphorylation of the signalling complex

If titin kinase is indeed a pseudokinase, it is necessary to consider an alternate mechanism for the phosphorylation and activation of SRF. One possibility is that nbr1 phosphorylation increases via phosphate transfer from glycogen synthase kinase 3 (GSK3)[148], which in turn in known to be phosphorylated by AKT during exercise[149]. Note that the phosphorylation of nbr1 could then occur after the formation of the mechanosensing complex. If the mechanosensor lasts a sufficiently long time, which we know it must from the nuclear translocation dynamics of MuRF, then we can assume that the phosphorylation of nbr1 and phosphate transfer occurs faster than the degradation time of the complex. Under normal physiological conditions therefore, it should be possible to ignore the phosphorylation rates of nbr1 as well as the rates of p62 and MuRF binding: the key step is the binding to nbr1 before the TK closes again under force. After nbr1 is bound, TK cannot close and the complex can fully aggregate.

REFERENCES

[1] Sadayappan S, Gulick J, Osinska H, Martin L A, Hahn H S, Dorn G W, Klevitsky R, Seidman C E, Seidman J G and Robbins J 2005 *Circ. Res.* **97** 1156–1163 ISSN 0009-7330

[2] Karsai Á, Kellermayer M S and Harris S P 2011 *Biophys. J.* **101** 1968–1977 ISSN 00063495

[3] Craig R and Offer G 1976 *Proc. R. Soc. London. Ser. B. Biol. Sci.* **192** 451–461 ISSN 0080-4649

[4] Bennett P, Craig R, Starr R and Offer G 1986 *J. Muscle Res. Cell Motil.* **7** 550–567 ISSN 0142-4319

[5]Offer G, Moos C and Starr R 1973 *J. Mol. Biol.* **74** 653–676 ISSN 00222836
[6]Knöll R 2012 *J. Muscle Res. Cell Motil.* **33** 31–42 ISSN 0142-4319
[7]Moss R L, Fitzsimons D P and Ralphe J C 2015 *Circ. Res.* **116** 183–192 ISSN 0009-7330
[8]McNamara J W, Singh R R and Sadayappan S 2019 *Proc. Natl. Acad. Sci.* 201821660 ISSN 0027-8424
[9]Higuchi H, Yanagida T and Goldman Y 1995 *Biophys. J.* **69** 1000–1010 ISSN 00063495
[10]Gautel M 2011 *Pflügers Arch. - Eur. J. Physiol.* **462** 119–134 ISSN 0031-6768
[11]Agarkova I and Perriard J C 2005 *Trends Cell Biol.* **15** 477–485 ISSN 09628924
[12]Pinotsis N, Chatziefthimiou S D, Berkemeier F, Beuron F, Mavridis I M, Konarev P V, Svergun D I, Morris E, Rief M and Wilmanns M 2012 *PLoS Biol.* **10** e1001261 ISSN 1545-7885
[13]Luther P K 2009 *J. Muscle Res. Cell Motil.* ISSN 01424319
[14]Gautel M and Djinović-Carugo K 2016 *J. Exp. Biol.* **219** 135–145 ISSN 0022-0949
[15]Burkholder T J 2007 *Front. Biosci.* **12** 174 ISSN 10939946
[16]Frank D, Kuhn C, Katus H A and Frey N 2006 *J. Mol. Med.* **84** 446–468 ISSN 0946-2716
[17]Frank D and Frey N 2011 *J. Biol. Chem.* **286** 9897–9904 ISSN 0021-9258
[18]Krüger M and Kötter S 2016 *Front. Physiol.* **7** ISSN 1664-042X
[19]Knöll R, Hoshijima M, Hoffman H M, Person V, Lorenzen-Schmidt I, Bang M L, Hayashi T, Shiga N, Yasukawa H, Schaper W, McKenna W, Yokoyama M, Schork N J, Omens J H, McCulloch A D, Kimura A, Gregorio C C, Poller W, Schaper J, Schultheiss H P and Chien K R 2002 *Cell* **111** 943–955 ISSN 00928674
[20]Knöll R, Linke W A, Zou P, Miočič S, Kostin S, Buyandelger B, Ku C H, Neef S, Bug M, Schäfer K, Knöll G, Felkin L E, Wessels J, Toischer K, Hagn F, Kessler H, Didié M, Quentin T, Maier L S, Teucher N, Unsöld B, Schmidt A, Birks E J, Gunkel S, Lang P, Granzier H, Zimmermann W H, Field L J, Faulkner G, Dobbelstein M, Barton P J, Sattler M, Wilmanns M and Chien K R 2011 *Circ. Res.* **109** 758–769 ISSN 0009-7330
[21]Witt C C, Burkart C, Labeit D, McNabb M, Wu Y, Granzier H and Labeit S 2006 *EMBO J.* **25** 3843–3855 ISSN 0261-4189
[22]Wakabayashi K, Sugimoto Y, Tanaka H, Ueno Y, Takezawa Y and Amemiya Y 1994 *Biophys. J.* **67** 2422–2435 ISSN 00063495
[23]Nunes J P, Schoenfeld B J, Nakamura M, Ribeiro A S, Cunha P M and Cyrino E S 2020 *Clin. Physiol. Funct. Imaging* **40** 148–156 ISSN 1475-0961
[24]Linke W A, Ivemeyer M, Mundel P, Stockmeier M R and Kolmerer B 1998 *Proc. Natl. Acad. Sci.* **95** 8052–8057 ISSN 0027-8424
[25]Linke W A, Pollack, Linke, Trombitás, Granzier, Labeit, TerKeurs, Rief, Bullard and Baatsen 2000 Titin elasticity in the context of the sarcomere: Force and extensibility measurements on single myofibrils *Adv. Exp. Med. Biol.* ISSN 00652598
[26]Bianco P, Mártonfalvi Z, Naftz K, Kőszegi D and Kellermayer M 2015 *Biophys. J.* **109** 340–345 ISSN 00063495
[27]Lange S, Pinotsis N, Agarkova I and Ehler E 2020 *Biochim. Biophys. Acta - Mol. Cell Res.* **1867** 118440 ISSN 01674889
[28]Schoenauer R, Bertoncini P, Machaidze G, Aebi U, Perriard J C, Hegner M and Agarkova I 2005 *J. Mol. Biol.* **349** 367–379 ISSN 00222836
[29]Mayans O, van der Ven P F M, Wilm M, Mues A, Young P, Fürst D O, Wilmanns M and Gautel M 1998 *Nature* **395** 863–869 ISSN 0028-0836
[30]Mitra S K, Hanson D A and Schlaepfer D D 2005 *Nat. Rev. Mol. Cell Biol.* **6** 56–68 ISSN 1471-0072
[31]Mitra S K and Schlaepfer D D 2006 *Curr. Opin. Cell Biol.* **18** 516–523 ISSN 09550674
[32]Bell S and Terentjev E M 2017 *Biophys. J.* **112** 2439–2450 ISSN 00063495
[33]Huxley H 1968 *J. Mol. Biol.* **37** 507–520 ISSN 00222836
[34]Rome E 1972 *J. Mol. Biol.* **65** 331–345 ISSN 00222836
[35]Haselgrove J C 2011 Structure of Vertebrate Striated Muscle as Determined by X-ray-Diffraction Studies *Compr. Physiol.* (Hoboken, NJ, USA: John Wiley & Sons, Inc.)
[36]MILLMAN B M 1998 *Physiol. Rev.* **78** 359–391 ISSN 0031-9333
[37]Krivickas L S, Dorer D J, Ochala J and Frontera W R 2011 *Exp. Physiol.* **96** 539–547 ISSN 09580670
[38]Kubo K, Ikebukuro T, Yata H, Tsunoda N and Kanehisa H 2010 *J. Strength Cond. Res.* **24** 322–331 ISSN 1064-8011
[39]Hu Z, Taylor D W, Edwards R J and Taylor K A 2017 *J. Struct. Biol.* **200** 334–342 ISSN 10478477
[40]Ma W, Gong H, Kiss B, Lee E J, Granzier H and Irving T 2018 *Biophys. J.* **115** 1580–1588 ISSN 00063495
[41]Åström E, Friman G and Pilström L 1977 *Ups. J. Med. Sci.* **82** 191–194 ISSN 0300-9734
[42]Capitanio M, Canepari M, Maffei M, Beneventi D, Monico C, Vanzi F, Bottinelli R and Pavone F S 2012 *Nat. Methods* **9** 1013–1019 ISSN 1548-7091
[43]Uyeda T Q, Kron S J and Spudich J A 1990 *J. Mol. Biol.* **214** 699–710 ISSN 00222836
[44]Walcott S, Warshaw D M and Debold E P 2012 *Biophys. J.* **103** 501–510 ISSN 00063495
[45]Persson M, Bengtsson E, ten Siethoff L and Månsson A 2013 *Biophys. J.* **105** 1871–1881 ISSN 00063495
[46]Rastogi K, Puliyakodan M S, Pandey V, Nath S and Elangovan R 2016 *Sci. Rep.* **6** 32043 ISSN 2045-2322
[47]Harris D E and Warshaw D M 1993 *J. Biol. Chem.* **268** 14764–14768 ISSN 00219258
[48]O'Connell C B, Tyska M J and Mooseker M S 2007 *Biochim. Biophys. Acta - Mol. Cell Res.* **1773** 615–630 ISSN 01674889
[49]HUXLEY A F and SIMMONS R M 1971 *Nature* **233** 533–538 ISSN 0028-0836
[50]Piazzesi G and Lombardi V 1995 *Biophys. J.* ISSN 00063495
[51]Caremani M, Melli L, Dolfi M, Lombardi V and Linari M 2013 *J. Physiol.* **591** 5187–5205 ISSN 00223751
[52]Caremani M, Melli L, Dolfi M, Lombardi V and Linari M 2015 *J. Physiol.* **593** 3313–3332 ISSN 00223751
[53]Holmes K C and Geeves M A 2000 *Philos. Trans. R. Soc. London. Ser. B Biol. Sci.* **355** 419–431 ISSN 0962-8436
[54]Mijailovich S M, Prodanovic M and Irving T C 2019 *Int. J. Mol. Sci.* **20** 6044 ISSN 1422-0067
[55]Tsaturyan A K, Bershitsky S Y, Koubassova N A, Fernandez M, Narayanan T and Ferenczi M A 2011 *Biophys. J.* **101** 404–410 ISSN 00063495
[56]Kellermayer M, Sziklai D, Papp Z, Decker B, Lakatos E and Mártonfalvi Z 2018 *J. Struct. Biol.* **203** 46–53 ISSN 10478477
[57]Reconditi M, Fusi L, Caremani M, Brunello E, Linari M, Piazzesi G, Lombardi V and Irving M 2019 *Biophys. J.* **116** 983–984 ISSN 00063495
[58]Puchner E M, Alexandrovich A, Kho A L, Hensen U, Schafer L V, Brandmeier B, Grater F, Grubmuller H, Gaub H E and Gautel M 2008 *Proc. Natl. Acad. Sci.* **105** 13385–13390 ISSN 0027-8424

[59]Gräter F, Shen J, Jiang H, Gautel M and Grubmüller H 2005 *Biophys. J.* **88** 790–804 ISSN 00063495 URL https://pubmed.ncbi.nlm.nih.gov/15531631/
[60]Tskhovrebova L, Trinick J, Sleep J A and Simmons R M 1997 *Nature* **387** 308–312 ISSN 0028-0836
[61]Linke W A, Kulke M, Li H, Fujita-Becker S, Neagoe C, Manstein D J, Gautel M and Fernandez J M 2002 *J. Struct. Biol.* **137** 194–205 ISSN 10478477
[62]Leake M C, Wilson D, Gautel M and Simmons R M 2004 *Biophys. J.* **87** 1112–1135 ISSN 00063495
[63]Rief M 1997 *Science (80-.).* **276** 1109–1112 ISSN 00368075
[64]Bustamante C, Marko J, Siggia E and Smith S 1994 *Science (80-.).* **265** 1599–1600 ISSN 0036-8075
[65]Biswas S, Leitao S, Theillaud Q, Erickson B W and Fantner G E 2018 *Sci. Rep.* **8** 9390 ISSN 20452322 URL www.nature.com/scientificreports
[66]Bullerjahn J T, Sturm S and Kroy K 2014 *Nat. Commun.* **5** 1–10 ISSN 20411723 URL www.nature.com/naturecommunications
[67]Zhou J, Aponte-Santamaría C, Sturm S, Bullerjahn J T, Bronowska A and Gräter F 2015 *PLoS Comput. Biol.* **11** ISSN 15537358 URL https://pubmed.ncbi.nlm.nih.gov/26544178/
[68]Lange S 2005 *Science (80-.).* **308** 1599–1603 ISSN 0036-8075
[69]The zinc-finger protein nbr1 binds forms a dimer via coiled-coil interactions[78,79]. nbr1 is known to bind to p62 and to titin kinase[68,150] via its PB1 domain. In other words, one PB1 domain must open for nbr1 to bind to titin kinase. There will be some degree of cooperativity between two nbr1 dimers binding to titin kinase. This will force the coefficient for the Hill equation to be between 1 (no cooperativity) and 2 (two nbr1 molecules must bind simultaneously to titin kinase) - see next paragraph.
[70]The maximum possible rate of signalling complex aggregation would occur if titin were bathing in a pool of nbr1, p62 and MuRF. Obviously this situation is non-physiological.
[71]Weiss J N 1997 *FASEB J.* **11** 835–841 ISSN 0892-6638
[72]Larsen K B, Lamark T, Øvervatn A, Harneshaug I, Johansen T and Bjørkøy G 2010 *Autophagy* **6** 784–793 ISSN 1554-8627
[73]Note that the faster degradation of nbr1 and p62 in starved cells might contribute to a reduction in hypertrophy during endurance exercise in which cells are starved, in a manner which is independent to chronic hypoxia[151] or AMPK signalling[152,153].
[74]Nowak M, Suenkel B, Porras P, Migotti R, Schmidt F, Kny M, Zhu X, Wanker E E, Dittmar G, Fielitz J and Sommer T 2019 *J. Cell Sci.* **132** jcs233395 ISSN 0021-9533
[75]Isaacs W B, Kim I S, Struve A and Fulton A B 1989 *J. Cell Biol.* **109** 2189–2195 ISSN 0021-9525
[76]Short B 2011 *J. Cell Biol.* **193** 597–597 ISSN 1540-8140
[77]Terry E E, Zhang X, Hoffmann C, Hughes L D, Lewis S A, Li J, Wallace M J, Riley L A, Douglas C M, Gutierrez-Monreal M A, Lahens N F, Gong M C, Andrade F, Esser K A and Hughes M E 2018 *Elife* **7** e34613 ISSN 2050-084X
[78]Kirkin V, Lamark T, Sou Y S, Bjørkøy G, Nunn J L, Bruun J A, Shvets E, McEwan D G, Clausen T H, Wild P, Bilusic I, Theurillat J P, Øvervatn A, Ishii T, Elazar Z, Komatsu M, Dikic I and Johansen T 2009 *Mol. Cell* **33** 505–516 ISSN 10972765
[79]Johansen T and Lamark T 2011 *Autophagy* **7** 279–296 ISSN 1554-8627
[80]Chen C S 2008 *J. Cell Sci.* **121** 3285–3292 ISSN 0021-9533
[81]Irving T, Wu Y, Bekyarova T, Farman G P, Fukuda N and Granzier H 2011 *Biophys. J.* **100** 1499–1508 ISSN 00063495
[82]ter Keurs H E, Iwazumi T and Pollack G H 1978 *J. Gen. Physiol.* **72** 565–592 ISSN 0022-1295
[83]Wang K 1985 Sarcomere-Associated Cytoskeletal Lattices in Striated Muscle *Cell Muscle Motil.* (Boston, MA: Springer US) pp 315–369
[84]Maruyama K 1986 Connectin, an Elastic Filamentous Protein of Striated Muscle *Int. Rev. Cytol.* (Academic Press Inc.) pp 81–114
[85]Trinick J 1991 *Curr. Opin. Cell Biol.* **3** 112–119 ISSN 09550674
[86]Sahlin K, Katz A and Henriksson J 1987 *Biochem. J.* **245** 551–556 ISSN 0264-6021
[87]CHANCE W T, CAO L and FISCHER J E 1988 *Ann. Surg.* **208** 524–531 ISSN 0003-4932
[88]Gokhin D S and Fowler V M 2013 *Nat. Rev. Mol. Cell Biol.* **14** 113–119 ISSN 1471-0072
[89]Egelman E H 2012 *Comprehensive Biophysics* (Elsevier) ISBN 9780080957180
[90]Alberts B, Johnson A, Lewis J, Morgan D, Raff M, Roberts K and Walter P 2017 *Molecular Biology of the Cell* (Garland Science) ISBN 9781315735368
[91]Melnikov S, Ben-Shem A, Garreau de Loubresse N, Jenner L, Yusupova G and Yusupov M 2012 *Nat. Struct. Mol. Biol.* **19** 560–567 ISSN 1545-9993
[92]Hammond D E, Claydon A J, Simpson D M, Edward D, Stockley P, Hurst J L and Beynon R J 2016 *Mol. Cell. Proteomics* **15** 1204–1219 ISSN 1535-9476
[93]Dennis P P and Bremer H 2008 *EcoSal Plus* **3** ISSN 2324-6200
[94]Kafri M, Metzl-Raz E, Jona G and Barkai N 2016 *Cell Rep.* **14** 22–31 ISSN 22111247
[95]Warner J R 1999 *Trends Biochem. Sci.* **24** 437–440 ISSN 09680004
[96]WARNER J, VILARDELL J and SOHN J 2001 *Cold Spring Harb. Symp. Quant. Biol.* **66** 567–574 ISSN 0091-7451
[97]Chaillou T, Kirby T J and McCarthy J J 2014 *J. Cell. Physiol.* **229** 1584–1594 ISSN 00219541
[98]Wen Y, Alimov A P and McCarthy J J 2016 *Exerc. Sport Sci. Rev.* **44** 110–115 ISSN 0091-6331
[99]Nakada S, Ogasawara R, Kawada S, Maekawa T and Ishii N 2016 *PLoS One* **11** e0147284 ISSN 1932-6203
[100]Fyfe J J, Bishop D J, Bartlett J D, Hanson E D, Anderson M J, Garnham A P and Stepto N K 2018 *Sci. Rep.* **8** 560 ISSN 2045-2322
[101]Brook M S, Wilkinson D J, Smith K and Atherton P J 2019 *Eur. J. Sport Sci.* **19** 952–963 ISSN 1746-1391
[102]von Walden F 2019 *J. Appl. Physiol.* **127** 591–598 ISSN 8750-7587
[103]MacDougall J D, Gibala M J, Tarnopolsky M A, MacDonald J R, Interisano S A and Yarasheski K E 1995 *Can. J. Appl. Physiol.* **20** 480–486 ISSN 1066-7814
[104]Biolo G, Tipton K D, Klein S and Wolfe R R 1997 *Am. J. Physiol. Metab.* **273** E122–E129 ISSN 0193-1849
[105]Hipp L, Beer J, Kuchler O, Reisser M, Sinske D, Michaelis J, Gebhardt J C M and Knöll B 2019 *Proc. Natl. Acad. Sci.* **116** 880–889 ISSN 0027-8424
[106]Ritter M, Wöll E, Häussinger D and Lang F 1992 *FEBS Lett.* **307** 367–370 ISSN 00145793
[107]French S L, Osheim Y N, Cioci F, Nomura M and Beyer A L 2003 *Mol. Cell. Biol.* **23** 1558–1568 ISSN 0270-7306
[108]Pérez-Ortín J E, Alepuz P M and Moreno J 2007 *Trends Genet.* **23** 250–257 ISSN 01689525
[109]KOBAYASHI T 2014 *Proc. Japan Acad. Ser. B* **90** 119–129 ISSN 0386-2208
[110]Stoykova A S, Dudov K P, Dabeva M D and Hadjiolov A A 1983 *J. Neurochem.* **41** 942–949 ISSN 0022-3042

[111] Yoshikado T, Toshimoto K, Nakada T, Ikejiri K, Kusuhara H, Maeda K and Sugiyama Y 2017 *Drug Metab. Dispos.* **45** 779–789 ISSN 0090-9556
[112] Nikolov E N, Dabeva M D and Nikolov T K 1983 *Int. J. Biochem.* **15** 1255–1260 ISSN 0020711X
[113] Mathis A D, Naylor B C, Carson R H, Evans E, Harwell J, Knecht J, Hexem E, Peelor F F, Miller B F, Hamilton K L, Transtrum M K, Bikman B T and Price J C 2017 *Mol. Cell. Proteomics* **16** 243–254 ISSN 1535-9476
[114] Ashford A J and Pain V M 1986 *J. Biol. Chem.* **261** 4066–70 ISSN 0021-9258
[115] Ashford A J and Pain V M 1986 *J. Biol. Chem.* **261** 4059–65 ISSN 0021-9258
[116] Dice J F, Dehlinger P J and Schimke R T 1973 *J. Biol. Chem.* **248** 4220–4228 ISSN 00219258
[117] Ohtsuki I, Maruyama K and Ebashi S 1986 Regulatory and Cytoskeletal Proteins of Vertebrate Skeletal Muscle *Adv. Protein Chem.* (Academic Press Inc.) pp 1–67
[118] Amos L A and Amos W B 1991 *Molecules of the Cytoskeleton* (London: Macmillan Education UK) ISBN 978-0-333-49595-7
[119] Marx J O, Kraemer W J, Nindl B C and Larsson L 2002 *Journals Gerontol. Ser. A Biol. Sci. Med. Sci.* **57** B232–B238 ISSN 1079-5006
[120] Martin A F 1981 *J. Biol. Chem.* **256** 964–8 ISSN 0021-9258
[121] Russell B, Motlagh D and Ashley W W 2000 *J. Appl. Physiol.* **88** 1127–1132 ISSN 8750-7587
[122] Toyama B H and Hetzer M W 2013 *Nat. Rev. Mol. Cell Biol.* **14** 55–61 ISSN 1471-0072
[123] Ross J F and Orlowski M 1982 *J. Bacteriol.* **149** 650–653 ISSN 0021-9193
[124] Rudolph F, Hüttemeister J, da Silva Lopes K, Jüttner R, Yu L, Bergmann N, Friedrich D, Preibisch S, Wagner E, Lehnart S E, Gregorio C C and Gotthardt M 2019 *Proc. Natl. Acad. Sci.* **116** 25126–25136 ISSN 0027-8424
[125] An extrapolation of titin mRNA diffusion times from[126] would indicate that titin mRNA effectively cannot diffuse through the cell: the diffusion coefficient for a globular protein of the same mass is almost zero due to the sieve-like nature of the myofibrillar lattice.
[126] Papadopoulos S, Jürgens K D and Gros G 2000 *Biophys. J.* **79** 2084–2094 ISSN 00063495
[127] Krzysztofik, Wilk, Wojdała and Gołaś 2019 *Int. J. Environ. Res. Public Health* **16** 4897 ISSN 1660-4601
[128] Baker J S, McCormick M C and Robergs R A 2010 *J. Nutr. Metab.* **2010** 1–13 ISSN 2090-0724
[129] Karlsson J, Nordesjö L O, Jorfeldt L and Saltin B 1972 *J. Appl. Physiol.* **33** 199–203 ISSN 8750-7587
[130] Achten J, Venables M C and Jeukendrup A E 2003 *Metabolism* **52** 747–752 ISSN 00260495
[131] Lehninger A L, Wadkins C L, Cooper C, Devlin T M and Gamble J L 1958 *Science (80-.).* ISSN 00368075
[132] Schatz G 1967 *Angew. Chemie Int. Ed. English* **6** 1035–1046 ISSN 0570-0833
[133] Murray B and Rosenbloom C 2018 *Nutr. Rev.* **76** 243–259 ISSN 0029-6643
[134] Kaya M, Tani Y, Washio T, Hisada T and Higuchi H 2017 *Nat. Commun.* **8** 16036 ISSN 2041-1723
[135] Woody M S, Winkelmann D A, Capitanio M, Ostap E M and Goldman Y E 2019 *Elife* **8** ISSN 2050-084X
[136] Kappenstein J, Ferrauti A, Runkel B, Fernandez-Fernandez J, Müller K and Zange J 2013 *Eur. J. Appl. Physiol.* **113** 2769–2779 ISSN 1439-6319
[137] McMahon S and Jenkins D 2002 *Sport. Med.* **32** 761–784 ISSN 0112-1642
[138] Harris R C, Edwards R H T, Hultman E, Nordesjö L O, Nylind B and Sahlin K 1976 *Pflögers Arch. Eur. J. Physiol.* **367** 137–142 ISSN 0031-6768
[139] Mendez-Villanueva A, Edge J, Suriano R, Hamer P and Bishop D 2012 *PLoS One* **7** e51977 ISSN 1932-6203
[140] Rich P 2003 *Biochem. Soc. Trans.* **31** 1095–1105 ISSN 0300-5127
[141] Rønnestad B R, Hansen J, Stensløkken L, Joyner M J and Lundby C 2019 *J. Appl. Physiol.* **127** 306–311 ISSN 8750-7587
[142] Janssen I, Heymsfield S B, Wang Z and Ross R 2000 *J. Appl. Physiol.* **89** 81–88 ISSN 8750-7587
[143] Ranković G, Mutavdžić V, Toskić D, Preljević A, Kocić M, Nedin-Ranković G and Damjanović N 2010 *Bosn. J. Basic Med. Sci.* **10** 44–48 ISSN 1840-4812
[144] Akagi R, Takai Y, Ohta M, Kanehisa H, Kawakami Y and Fukunaga T 2009 *Age Ageing* **38** 564–569 ISSN 0002-0729
[145] Waugh C M, Korff T, Fath F and Blazevich A J 2014 *J. Appl. Physiol.* **117** 257–266 ISSN 8750-7587
[146] Ettema G and Huijing P 1994 *J. Biomech.* **27** 1361–1368 ISSN 00219290
[147] Ateş F, Hug F, Bouillard K, Jubeau M, Frappart T, Couade M, Bercoff J and Nordez A 2015 *J. Electromyogr. Kinesiol.* **25** 703–708 ISSN 10506411
[148] Nicot A S, Lo Verso F, Ratti F, Pilot-Storck F, Streichenberger N, Sandri M, Schaeffer L and Goillot E 2014 *Autophagy* **10** 1036–1053 ISSN 1554-8627
[149] Hermida M A, Dinesh Kumar J and Leslie N R 2017 *Adv. Biol. Regul.* **65** 5–15 ISSN 22124926
[150] Lamark T, Perander M, Outzen H, Kristiansen K, Øvervatn A, Michaelsen E, Bjørkøy G and Johansen T 2003 *J. Biol. Chem.* **278** 34568–34581 ISSN 0021-9258
[151] Deldicque L and Francaux M 2013 *Cell. Mol. Exerc. Physiol.* **2** ISSN 2049-419X
[152] Mounier R, Lantier L, Leclerc J, Sotiropoulos A, Foretz M and Viollet B 2011 *Cell Cycle* **10** 2640–2646 ISSN 1538-4101
[153] Thomson D 2018 *Int. J. Mol. Sci.* **19** 3125 ISSN 1422-0067
